# Gut microbiota-immune-bone axis in osteoporosis: mechanisms and intervention strategies

**DOI:** 10.3389/fphar.2026.1879472

**Published:** 2026-10-09

**Authors:** Minying Huang, Baocheng Xie, Hongyu Liu

**Affiliations:** 1 Department of Pharmacy, Guangdong Provincial Key Laboratory of Major Obstetric Diseases; Guangdong Provincial Clinical Research Center for Obstetrics and Gynecology; The Third Affiliated Hospital, Guangzhou Medical University, Guangzhou, China; 2 Department of Pharmacy, The Tenth Affiliated Hospital, Southern Medical University (Dongguan People’s Hospital), Dongguan, Guangdong, China; 3 Department of Pharmacy, Central Hospital of Guangdong Prison, Guangzhou, China

**Keywords:** gut microbiota, gut-immune-bone axis, immunity, osteoimmunology, osteoporosis, probiotics

## Abstract

Osteoporosis (OP) remains a major global health burden, yet current pharmacological interventions are constrained by adverse effects, limited efficacy, and a bone-centric therapeutic paradigm that overlooks upstream pathogenic drivers. Emerging evidence positions the gut microbiota (GM) as a critical rheostat of bone homeostasis, operating through a gut–immune–bone axis that integrates microbial metabolism, intestinal barrier integrity, and systemic immune regulation. This review systematically examines the immunopathological mechanisms of OP, with particular emphasis on the GM-derived metabolites—short-chain fatty acids, secondary bile acids, tryptophan derivatives, lipopolysaccharide, and trimethylamine N-oxide—that orchestrate osteoclastogenic versus osteoprotective outcomes via key immune checkpoints including the Th17/Treg, macrophage phenotypic polarization, and regulatory B cell axes. We further delineate the subtype-specific heterogeneity of this axis across postmenopausal, senile, glucocorticoid-induced, and diabetic osteoporosis, highlighting distinct microbial and immune signatures that mandate stratified therapeutic approaches. Importantly, we evaluate the translational potential of GM-targeted interventions—probiotics, prebiotics, fecal microbiota transplantation, and dietary modulation—as adjunctive or standalone strategies, and discuss their synergistic interplay with conventional anti-osteoporotic agents. By reframing OP as a systems-level disorder of the gut–immune–bone circuitry, this review establishes a mechanistic groundwork for precision medicine, identifies novel druggable targets, and charts a course toward safer, more durable, and disease-modifying anti-osteoporotic therapies.

## 1 Introduction

Osteoporosis (OP) is a systemic metabolic skeletal disorder characterized by diminished bone strength, reduced bone mass, and microarchitectural deterioration, culminating in increased bone fragility and fracture susceptibility (Ye et al., 2025). As a major global health burden, OP affects hundreds of millions worldwide, with approximately 178 million new fragility fractures documented in 2019 alone (Wu et al., 2021). With the accelerating aging population, the prevalence of OP and its associated socioeconomic costs are projected to escalate dramatically, underscoring an urgent need for more effective prevention and therapeutic strategies.

Historically, research on OP pathogenesis has predominantly centered on bone-intrinsic mechanisms—including aberrant mesenchymal stem cell differentiation, estrogen deficiency, oxidative stress, and chronic inflammation—while the pivotal role of the immune system in orchestrating bone metabolism has been comparatively underexplored (Li et al., 2025; Srivastava et al., 2018; Wang et al., 2022b). This perspective shifted markedly in 2000 when Takayanagi et al. (2000) formally introduced the concept of “osteoimmunology,” establishing a conceptual framework for the bidirectional crosstalk between the immune and skeletal systems. Subsequent investigations have reaffirmed that these two systems share a vast repertoire of signaling molecules, including cytokines, chemokines, hormones, and their cognate receptors (Nusrat et al., 2025). Immune cells are now recognized as deeply embedded regulators of every phase of bone remodeling: neutrophils recruit mesenchymal stem cells and secrete inflammatory mediators (Kovtun et al., 2016); macrophages, through polarization toward pro-inflammatory (“M1-like”) or reparative (“M2-like”) states, exert opposing effects on osteogenesis and osteoclastogenesis (Almeida et al., 2016; Vallés et al., 2020); and T and B lymphocytes modulate the RANKL/OPG axis to fine-tune osteoclast activity (Li et al., 2007). Thus, unraveling the immunopathological underpinnings of OP is essential for a comprehensive understanding of its pathogenesis.

Despite these insights, current pharmacotherapies for OP—including bisphosphonates, selective estrogen receptor modulators, calcitonin, and monoclonal antibodies—remain largely bone-centric, targeting terminal effectors of bone turnover rather than upstream drivers (Table 1). Bisphosphonates, for instance, can modulate the Th17/Treg balance, yet long-term use disrupts intestinal microecology and perpetuates low-grade inflammation (Badros et al., 2021). Teriparatide, a classic anabolic agent, expands bone marrow Tregs to facilitate Wnt10b-mediated osteogenesis, but its efficacy is entirely contingent on intact gut microbial metabolism and short-chain fatty acids (SCFAs) production (Li et al., 2020). Denosumab effectively suppresses osteoclastogenesis but fails to correct systemic inflammation driven by gut dysbiosis, and its withdrawal triggers rapid rebound bone loss accompanied by elevated intestinal inflammatory mediators (Khafipour et al., 2020; Sköld et al., 2022). These limitations highlight a critical therapeutic gap: conventional agents alleviate bone metabolic derangements without rectifying the upstream GM dysbiosis and consequent immune imbalance [e.g., aberrant Th17/Treg imbalances and macrophage polarization toward pro-inflammatory (“M1-like”) phenotypes].

**TABLE 1:** Current drugs for OP treatment and their limitations.

Classification	Drug subcategory	Representative drugs	Mechanism of action	Clinical efficacy	Main limitations	References
Anti-resorptive Drugs	Bisphosphonates	Alendronate, Risedronate, Zoledronic acid	Inhibit OC activity and bone resorption	Significantly increase lumbar spine and hip BMD; reduce the risk of vertebral and non-vertebral fractures	Gastrointestinal intolerance; contraindicated in severe renal insufficiency; long-term use associated with osteonecrosis of the jaw (ONJ) and atypical femoral fracture; poor long-term oral adherence	Black et al. (2020)
	Anti-RANKL Monoclonal Antibody	Denosumab	Bind and neutralize RANKL, thereby suppressing osteoclastogenesis and maturation	Rapidly reduces bone turnover; effective in patients intolerant to bisphosphonates	Rebound bone loss and increased fracture risk after discontinuation; hypocalcemia and infection risk; requires subcutaneous injection; high cost; limited long-term safety data	Kendler et al. (2022)
	Selective Estrogen Receptor Modulators (SERMs)	Raloxifene	Exerts estrogen-like protective effects on bone without stimulating breast or endometrial tissue	Reduces the risk of vertebral fractures; no increased risk of breast cancer	No significant effect on non-vertebral fractures; increased risk of venous thromboembolism; aggravates menopausal hot flashes; poor tolerance in some patients	Adomaityte et al. (2008)
	Hormone Replacement Therapy (HRT)	Conjugated estrogens, Estradiol	Suppresses bone resorption through estrogen receptor-mediated pathways	Effectively prevents postmenopausal bone loss	Increased risk of breast cancer, endometrial cancer, and cardiovascular events; limited applicable population; not recommended for long-term routine use	Nierengarten (2024)
	Calcitonin	Salmon calcitonin	Inhibits OC function and relieves osteoporotic bone pain	Rapidly alleviates osteoporotic bone pain; exerts a mild anti-resorptive effect	Weak long-term fracture prevention effect; increases the risk of cancer; easy to develop drug resistance; risk of allergic reactions; local nasal irritation with nasal spray formulation	Overman et al. (2013)
Anabolic Drugs	PTH Analogs	Teriparatide	Stimulates osteoblast proliferation and bone formation	Strong anabolic effect; particularly effective in severe OP	Usage duration limited to ≤2 years; potential osteosarcoma risk (warning from animal experiments); requires daily subcutaneous injection; high price; contraindicated in patients with a history of bone tumors	Gilsenan et al. (2020)
	Anti-SOST Antibody	Romosozumab	Inhibits SOST, activates the Wnt/β-catenin pathway, exerting dual anabolic and anti-resorptive effects	Rapid improvement in BMD; superior reduction in vertebral and non-vertebral fracture risks	Increased risk of cardiovascular events (myocardial infarction, stroke) in some clinical trials; only for short-term use (1–2 years); requires injection; high cost; contraindicated in patients with high cardiovascular risk	Fusaro and Tripepi (2023)
Basic Adjuvant Drugs	Calcium + Vitamin D	Calcium carbonate/citrate, Vitamin D3	Provides bone mineral raw materials; regulates calcium and phosphorus metabolism	Serves as fundamental adjuvant therapy; prevents bone loss and reduces the risk of secondary OP	Cannot independently reverse severe OP; excessive intake leads to hypercalcemia and kidney stones; weak anti-fracture effect when used alone	Alexander et al. (2022)
Novel Molecular Targeted Drugs (Investigational)	Cathepsin K Inhibitor	Odanacatib	Selectively inhibits cathepsin K to suppress OC-mediated bone resorption	Significant increase in BMD; convenient once-weekly oral administration	Increased risk of stroke (leading to termination of clinical development); skin adverse events; no longer clinically available	McClung et al. (2019)
	Anti-RANKL Monoclonal Antibody (Novel)	Narlumosbart (JMT103)	Fully-humanized anti-RANKL monoclonal antibody; blocks RANKL-RANK binding to inhibit osteoclastogenesis and activity	Hase II trials showed non-inferior efficacy to denosumab; 4.61%–6.40% lumbar BMD increase at 12 months vs. baseline; Phase III clinical trials not yet completed	Long-term safety data insufficient; currently approved only for giant cell tumor of bone, not for OP	Chi et al. (2025)

In this context, the gut microbiota (GM) has emerged as a key rheostat of bone homeostasis, operating through a “gut-bone” axis that integrates microbial metabolism, intestinal barrier integrity, and systemic immunity (An et al., 2025; De Vos et al., 2022). As the largest microecosystem in the human body, the GM interacts bidirectionally with the gastrointestinal, immune, endocrine, and nervous systems, influencing cellular responses across multiple organs (De Vos et al., 2022). Compelling evidence now indicates that GM and its metabolites not only directly modulate osteoblast and osteoclast activity but also exert profound regulatory effects via the Th17/Treg immune checkpoint—a nexus that maintains the balance between bone formation and resorption (Zhu et al., 2024). This tripartite circuitry, termed the “gut-immune-bone axis,” provides a mechanistic framework that reconciles microbial ecology, immune surveillance, and skeletal homeostasis, opening innovative avenues for OP prevention and treatment.

Herein, we systematically review the roles of immune cells and their cytokines in OP pathogenesis, delineate the regulatory effects of GM and its metabolites on bone metabolism, and elucidate the mechanistic architecture of the gut-immune-bone axis. We further evaluate emerging intervention strategies—including probiotics, prebiotics, fecal microbiota transplantation, and dietary modulation—and discuss their potential as adjunctive or standalone therapies. By reframing OP as a systems-level disorder of the gut-immune-bone circuitry, this review aims to establish a mechanistic groundwork for precision medicine and to chart a course toward safer, more durable, and disease-modifying anti-osteoporotic therapies.

## 2 Methods

This review followed a pre-established protocol with systematic literature searches conducted on 12 April 2026 across Web of Science, PubMed, Embase and CNKI using Boolean search string: (“Gut microbiota” OR “Gut microflora” OR “Gastrointestinal microbiome” OR “Microbiome, Gastrointestinal” OR “Enteric microbiota” OR “Intestinal flora”) AND (“Osteoporosis” OR “Osteoporosis, Age-Related” OR “Age-Related Osteoporosis” OR “Bone Loss, Age-Related” OR “Bone Losses, Age-Related” OR “Osteoporosis, Senile” OR “Senile Osteoporosis”) AND (“Immunity” OR “Immune Response” OR “Immune Responses” OR “Immune Process” OR “Immune Processes”). Studies investigating gut-immune-bone axis mechanisms and GM-targeted anti-osteoporotic interventions were included, whereas irrelevant articles, conference abstracts, duplicates, and incomplete datasets were excluded. Two independent reviewers performed deduplication, title/abstract and full-text screening, settling disagreements through discussion or a third investigator; 764 initial records were retrieved, and 78 qualified articles were finally enrolled, with full methodological details provided in Supplementary Material.

## 3 Osteoimmunology: the immune regulatory network in OP

The immune system profoundly influences bone metabolism, establishing the conceptual framework of osteoimmunology (Takayanagi et al., 2000), and the term “immunoporosis” was later introduced to emphasize the pathogenic role of immune cells in OP (Srivastava and Sapra, 2022). Within the bone marrow microenvironment, immune cells—particularly T cells and macrophages—engage in bidirectional crosstalk with osteoblasts (OBs) and osteoclasts (OCs) via shared cytokines and receptors, thereby orchestrating bone remodeling and homeostasis (Wang Y.-T. et al., 2025). For instance, T cell-derived TNF-α promotes OB apoptosis, B cell-produced RANKL drives osteoclastogenesis, and Th17-secreted IL-17 exerts dual effects—inducing osteogenic differentiation under certain conditions while promoting bone resorption in inflammatory contexts. As core effectors of the bone-immune network, immune cells secrete a diverse array of cytokines that directly or indirectly modulate OB/OC differentiation, function, and survival, playing a pivotal role in the onset and progression of OP (Figure 1). The key immune-related factors and cell-specific mechanisms are summarized in Tables 2 and 3, respectively, and the following subsections systematically review the regulatory actions of distinct immune cell subsets on bone metabolism.

**FIGURE 1:**
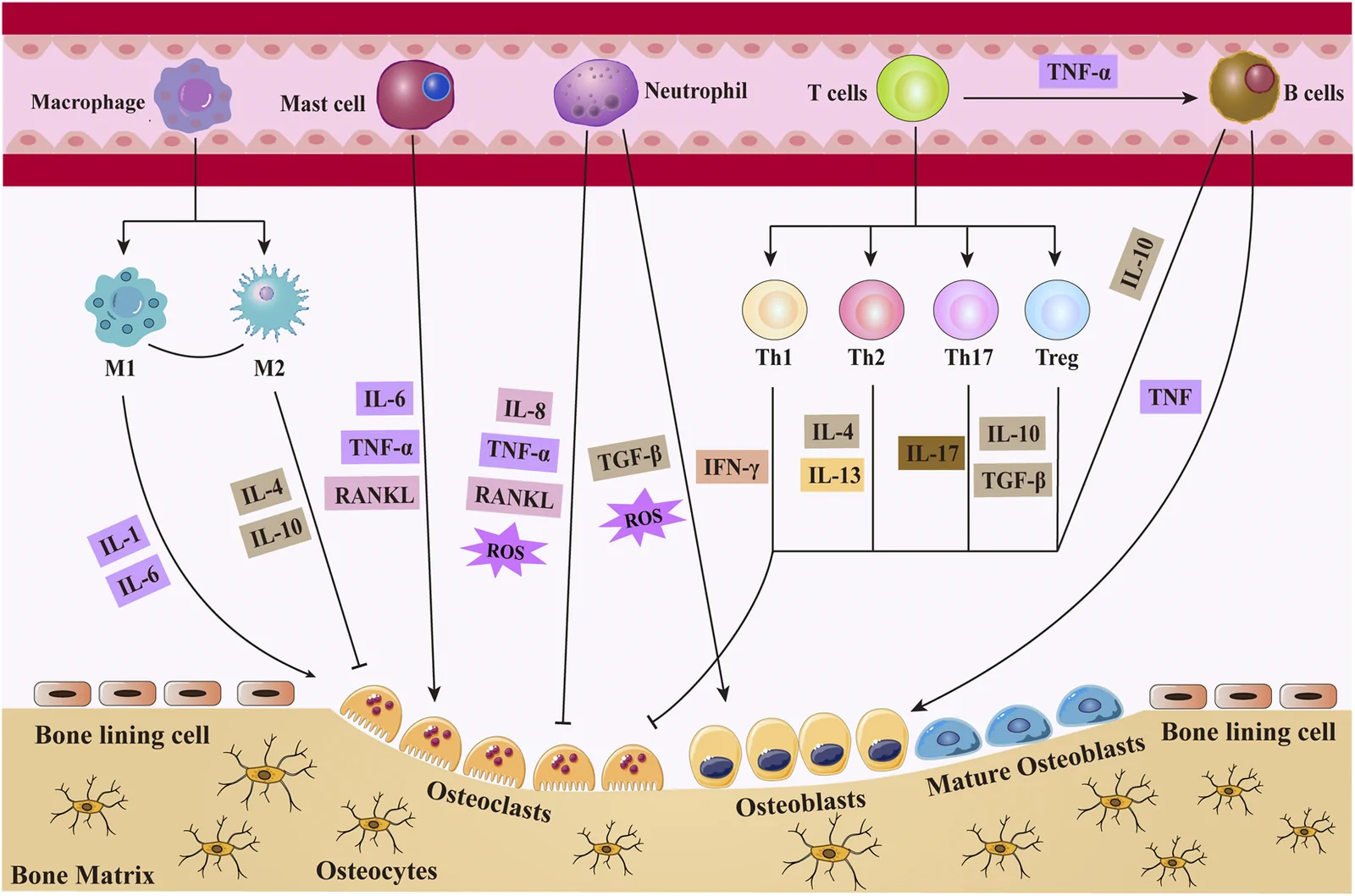
The Immune–bone regulatory network in OP. In the context of OP, various immune cells (T cells, B cells, macrophages, neutrophils, mast cells, etc.) directly or indirectly regulate OBs, OCs, and osteocytes by secreting key cytokines (TNF-α, IL-6, IL-17, IL-10, IFN-γ, TGF-β, etc.) to directly or indirectly regulate the differentiation and function of OBs, OCs, and osteocytes, thereby mediating an imbalance between bone formation and resorption. Together, these cells constitute the immune-bone regulatory network underlying the development of OP.

**TABLE 2:** Key cytokines governing immune-bone crosstalk in OP.

Cytokine	Role in immune response	Mechanism in OP	Effect on bone remodeling	References
IL-1	Pro-inflammatory cytokine that activates macrophages/neutrophils and amplifies inflammatory cascades	Promotes osteoclastogenesis, enhances bone resorption, and upregulates RANKL expression; biphasic osteoblastic effects via BMP/Smad pathway at specific concentrations	Dual (pro-resorptive dominant)	Tseng et al. (2022)
IL-4	An anti-inflammatory cytokine that modulates Th2 immunity and suppresses excessive inflammation	Inhibits OC formation and bone resorption; enhances osteoblast differentiation and OPG expression to balance the RANKL/OPG ratio	Bone-protective (anti-osteoporotic)	Muñoz et al. (2020)
IL-5	Th2 signature anti-inflammatory cytokine; promotes eosinophil maturation and recruitment, cooperates with IL-4/IL-13 to limit Th1/Th17 inflammatory responses	Suppresses RANKL-triggered osteoclast precursor differentiation; elevates OPG secretion from OBs and stromal cells to reduce net bone resorption	Bone-protective (anti-osteoporotic)	Jin et al. (2023)
IL-6	Dual-function cytokine with pro-inflammatory and immunomodulatory roles, regulating T/B cell activation	Promotes osteoclastogenesis and bone resorption via the RANKL/RANK/OPG pathway; may exert beneficial effects under physiological conditions (e.g., mechanical loading)	Pro-resorptive (pro-osteoporotic)	Wang and He (2020)
IL-8	Chemokine-like pro-inflammatory cytokine; recruits neutrophils, monocytes and Th17 cells to inflammatory sites, amplifies local inflammatory response	Indirectly boosts osteoclast formation by stimulating TNF-α and IL-17 release from immune cells; augments RANKL expression in stromal cells and OBs to accelerate bone breakdown	Pro-resorptive (pro-osteoporotic)	Rechenberg et al. (2014)
IL-9	Pleiotropic Th2/Treg-derived cytokine; regulates mast cell activation, limits Th17 overreaction and restrains exaggerated systemic inflammation	Dual regulatory function: low physiological levels suppress NFATc1 to block osteoclast maturation; high pathological concentrations trigger mast cell degranulation to release pro-resorptive mediators	Dual (bone-protective at physiological doses, pro-resorptive under severe inflammation)	Ting et al. (2018)
IL-10	Key anti-inflammatory cytokine that suppresses pro-inflammatory cytokine release and maintains immune tolerance	Inhibits osteoclastogenesis and maturation; upregulates OPG and downregulates RANKL to attenuate excessive bone resorption	Bone-protective (anti-osteoporotic)	Wang, Y. et al. (2020)
IL-12	Pro-inflammatory master cytokine secreted by dendritic cells and macrophages; drives Th1 cell differentiation, boosts IFN-γ secretion by T/NK cells, restrains Th17 polarization	Indirectly suppresses osteoclast formation through inducing IFN-γ to degrade TRAF6; chronic sustained IL-12 overproduction exacerbates inflammation and secondary bone loss via elevated TNF-α/IL-17; blunts RANKL-mediated osteoclast maturation	Dual (anti-resorptive dominant under acute inflammation; pro-resorptive under chronic inflammatory states)	Hideki et al. (2011)
IL-13	Th2-derived signature anti-inflammatory cytokine; functionally redundant with IL-4, promotes macrophage polarization toward a reparative (“M2-like”) phenotype, suppresses Th1/Th17 pro-inflammatory cascades, and inhibits the release of pro-inflammatory mediators from innate immune cells	Inhibits RANKL-induced osteoclast differentiation and bone resorption by downregulating RANKL expression and upregulating OPG secretion from OBs/stromal cells; blocks NF-κB signaling to reduce pro-resorptive cytokine release; promotes osteoblast proliferation and differentiation to enhance bone formation capacity	Bone-protective (anti-osteoporotic)	Shapouri-Moghaddam et al. (2018)
IL-15	Multifunctional pro-inflammatory cytokine secreted by monocytes, macrophages, dendritic cells and stromal cells; promotes proliferation, survival and activation of CD8^+^ T cells and NK cells, maintains memory CD8^+^ T cell homeostasis, synergizes with IL-12 to enhance IFN-γ secretion, and regulates Th1/Th17 immune responses	Indirectly promotes osteoclastogenesis and bone resorption by stimulating the release of pro-resorptive cytokines (TNF-α, IL-17) from immune cells; directly acts on osteoclast precursors to amplify RANKL-induced osteoclast differentiation via activating NF-κB and MAPK signaling pathways; inhibits osteoblast proliferation and differentiation, thus reducing bone formation capacity; sustained high expression of IL-15 under chronic inflammatory conditions exacerbates bone loss and OP progression	Pro-resorptive (pro-osteoporotic)	Okabe et al. (2017)
IL-17	Pro-inflammatory cytokine driving Th17-mediated immunity and recruiting inflammatory cells	Upregulates RANKL expression, shifts the RANKL/OPG ratio toward bone resorption, and directly promotes osteoclastogenesis	Pro-resorptive (pro-osteoporotic)	DeSelm et al. (2012)
IL-31	Pro-inflammatory; drives Th1/Th17 inflammation; suppresses Th2 protective responses	Activates STAT1/3/5, MAPK; induces IL-6, TNF-α, MMPs; promotes OC formation	Pro-resorptive (pro-osteoporotic)	De Martinis et al. (2020)
IL-33	Alarmin; drives Th2 immunity; induces Treg; inhibits NF-κB; suppresses Th17	Inhibits RANKL-dependent osteoclastogenesis; inactivates NFATc1; stimulates OB function	Bone-protective (anti-osteoporotic)	De Martinis et al. (2020)
TNF-α	Core pro-inflammatory cytokine that activates NF-κB signaling and mediates systemic inflammation	Activates RANKL to promote OC formation and inhibits OB function; exhibits biphasic effects (short-term low-concentration exposure may promote osteogenesis)	Pro-resorptive (pro-osteoporotic)	Zha et al. (2018)
IFN-γ	An immune-activating cytokine that modulates T/NK cell function and adaptive immunity	Inhibits osteoclastogenesis via TRAF6 degradation and Fas/FasL-mediated apoptosis; may impair osteogenesis under chronic inflammatory conditions	Dual (anti-resorptive dominant)	Shashkova et al. (2016)
IFN-β	Antiviral and immunomodulatory cytokine with anti-inflammatory feedback regulatory roles	Inhibits osteoclastogenesis by interfering with RANKL-induced c-Fos activation; acts as a negative feedback regulator to protect against excessive bone resorption	Bone-protective (anti-osteoporotic)	Zhao et al. (2014)
TGF-β	An immunomodulatory cytokine that balances Treg/Th17 responses and regulates immune cell differentiation	Biphasic role: promotes OC formation and inhibits OB differentiation at high concentrations; mediates OB proliferation and bone remodeling coupling under physiological conditions	Context-dependent (dual)	Travis and Sheppard (2013), Weivoda et al. (2016)

**TABLE 3:** Regulatory mechanisms of the immune cells in OP.

Immune cells	Subtype	Regulatory mechanism	Effects on bone metabolism	References
T lymphocytes	Th17	Secretes pro-inflammatory cytokines (e.g., TNF-α, IL-17, IL-6) via STAT3 signaling; activates RANKL and inhibits OPG.	Promote bone resorption, accelerate bone loss, aggravate OP	Wang et al. (2021c)
	Tregs	Secretes anti-inflammatory cytokines (e.g., IL-4, IL-10, TGF-β) via Foxp3 signaling; inhibits RANKL.	Inhibit bone resorption and preserve bone formation, maintain bone homeostasis, and reduce bone loss	Han et al. (2018)
B lymphocytes	B cells	↑ OPG production inhibits OB differentiation via ERK/NF-κB signaling; promotes OC differentiation via G-CSF secretion (in inflammatory conditions)	Mainly promotes bone resorption, involved in postmenopausal OP	Sun et al. (2018)
	Bregs	Secretes anti-inflammatory cytokines (e.g., IL-35, IL-10, TGF-β1)	Inhibit bone resorption, alleviate inflammatory bone loss	Li et al. (2023)
Macrophages	Pro-inflammatory polarization (“M1-like”)	Secretes pro-inflammatory cytokines/mediators (e.g., IL-1, IL-6, IL-12, TNF-α, NO, ROS)	Strongly promotes bone resorption, exacerbates bone destruction	Feng et al. (2019)
	Reparative polarization (“M2-like”)	Secretes anti-inflammatory cytokines (e.g., IL-10, TGF-β) and pro-osteogenic molecules (e.g., BMP-2)	Inhibit bone resorption, promote bone formation, and repair	Ling et al. (2025)
Dendritic cells (DCs)	Conventional DCs 1 (cDC1)	Highly expresses CD80/CD86 for antigen presentation; indirectly promotes osteoclastogenesis via T cell activation	Mainly inhibit OCs and preserve bone mass	Hoefsmit et al. (2023)
	Conventional DCs 2 (cDC2)	Suppresses NFATc1 nuclear translocation and osteoclastogenesis by promoting cdc2-mediated phosphorylation of NFATc1 at Ser263; downregulates osteoclastogenesis and TRAP activity	Promote bone resorption, participate in inflammatory bone loss	Kim, H.-M. et al. (2020)
Neutrophils	—	Mediates phagocytosis, degranulation, and neutrophil extracellular trap (NET) formation	Promote bone resorption, worsen bone microdamage, and bone loss	Isojima et al. (2025)
Mast cells	—	Modulates bone metabolism in the context of estrogen deficiency; it exerts pro-osteogenic effects via PDGF secretion	Promote bone resorption, involved in OP and bone pain	Ragipoglu et al. (2020)
Natural Killer cells	—	Secretes pro-osteogenic molecules (e.g., RANKL, M-CSF); IL-15-activated NK cells eliminate OCs via LFA-1/ICAM-1 and DNAM-1/CD155 interactions	Inhibit bone resorption, exert osteoprotective effects	Takeda et al. (2014)
Natural Killer T cells (NKT)	Invariant NKT (iNKT)	Activated by lipo-αGC; promotes Foxp3+ Treg expansion and proliferation in a BMC-dependent manner	Bidirectional effects: promote resorption in pro-inflammatory state, protect bone in anti-inflammatory state	Miyairi et al. (2017)
	CD56bright NKT	Enhances trafficking to inflammatory sites	Mainly osteoprotective, inhibit bone resorption, and maintain bone mineral density	Cunha et al. (2020)

### 3.1 T cells: central orchestrators of osteoimmune crosstalk

T cells originate from hematopoietic stem cells in the bone marrow and share a common precursor with OCs; they are key participants in immune responses and the regulation of bone metabolism. Mice with T-cell deficiencies exhibit enhanced osteoclastogenesis and reduced bone density, suggesting that T cells are crucial for skeletal health (Li et al., 2007). Upon stimulation by exogenous antigens, naive CD4^+^ T cells can differentiate into subsets such as Th17 cells, Tregs, Th1 cells, and Th2 cells. By secreting distinct cytokine profiles, these cells exert differential regulatory effects on bone metabolism (Buck et al., 2015; Zhu et al., 2010).

#### 3.1.1 Th17 cells: pro-inflammatory drivers of osteoclastogenesis

Th17 cells are a pro-inflammatory subset of CD4^+^ T cells; their activation not only exacerbates local inflammatory responses but also drives osteoclastogenesis and bone resorption activity (Ma et al., 2024). Studies (Srivastava et al., 2018) have shown that Th17 cell counts in the blood and peripheral tissues of patients with osteoarthritis (OA) are significantly higher than in healthy individuals, making them an important biomarker for OA. Th17 differentiation is regulated by the transcription factor retinoic acid-related orphan receptor γt (RORγt), which subsequently activates the expression of related genes and the secretion of pro-inflammatory factors such as IL-17 and IL-22 (Zhu et al., 2020).

IL-17, primarily secreted by Th17 cells, is a key mediator of their effect on bone. It upregulates inflammatory cytokines through the activation of multiple downstream signaling cascades (MAPK, ERK, and STAT3) (Kitaura et al., 2020). IL-17 promotes osteoclastogenesis by upregulating RANKL expression in OCs and synovial cells, thereby altering the RANKL/OPG ratio (Song et al., 2019; Tang et al., 2021). In rats with arthritis, IL-17 promotes the production of inflammatory factors and RANKL by regulating the IL-17/STAT3 signaling pathway (Ganesan and Rasool, 2017). IL-17 also enhances the autophagy activity of osteoclast precursor cells (OPCs) via the ERK/mTOR/Beclin-1 pathway, further promoting osteoclastogenesis (Tang et al., 2023). Furthermore, IL-17 induces macrophages to release inflammatory mediators such as TNF-α, IL-1, and IL-6, further amplifying the local inflammatory response and promoting bone resorption (Huang et al., 2022). Serum IL-17 levels are negatively correlated with bone density and may serve as a potential predictive marker for OP (Al-Qahtani et al., 2022). Studies (DeSelm et al., 2012; Tyagi et al., 2014) have shown that eliminating IL-17 or using anti-IL-17 antibodies (such as secukinumab) may prevent bone loss. Notably, IL-17 inhibits bone morphogenetic protein 2 (BMP-2) in OBs and induces pyroptosis via the NLRP3 inflammasome pathway (Lei et al., 2021). Thus, Th17 cells and the pro-inflammatory factors they secrete play a central driving role in bone metabolic imbalance.

#### 3.1.2 Tregs: immune guardians of bone homeostasis

Tregs are a key CD4^+^ T cell subset involved in maintaining immune tolerance and bone homeostasis; their function depends on the regulation of the surface marker CD25^+^ and the nuclear transcription factor forkhead box protein p3 (Foxp3) (Santamaria et al., 2021). Tregs suppress inflammatory responses by secreting IL-10 and TGF-β: IL-10 upregulates OPG and downregulates RANKL and M-CSF expression, thereby inhibiting osteoclastogenesis and maturation; TGF-β promotes Foxp3 expression and induces the conversion of conventional CD4^+^ T cells into Tregs (Lin et al., 2022; Wang et al., 2023). Although Foxp3^+^ CD8^+^ Treg cells do not affect OC survival, they can inhibit OC maturation and resorptive activity by suppressing actin ring formation (Shashkova et al., 2016). TGF-β secreted by Tregs can also activate the MAPK/Smad pathway, promoting OB proliferation and differentiation (Runyan et al., 2012). Other studies (Oh et al., 2010) have found that IL-35, a newly identified cytokine secreted by Treg cells, can suppress the expression of IL-17 to delay the progression of collagen-induced arthritis in mice. Overall, Treg cells inhibit bone resorption and promote bone formation through multiple mechanisms, and their numerical or functional deficiency constitutes an important immunological basis for OP.

#### 3.1.3 Th1 cells and Th2 cells: opposing modulators of bone resorption

Th1 cells differentiate from naïve CD4^+^ T cells under IL-12 stimulation. They secrete cytokines such as IFN-γ, IL-2, TNF-α, and GM-CSF, the majority of which suppress osteoclastogenesis (Raphael et al., 2015; Ren et al., 2017). IFN-γ released by activated T cells exerts a predominantly inhibitory effect on OCs: it suppresses early osteoclast formation while promoting the fusion of mononuclear OCs at later stages (Place et al., 2021). Studies (Wang et al., 2022a) have demonstrated that IFN-γ activates the JAK-STAT signaling pathway, particularly STAT1, which inhibits the expression of key transcription factors such as NFATc1 and c-Fos and blocks the transition of precursors into mature OCs. Even trace amounts of IFN-γ can inhibit osteoclastogenesis by inducing TRAF6 degradation. Additionally, IFN-γ may promote osteoblast formation but suppress bone marrow adipocyte formation. In contrast, TNF-α, also produced by Th1 cells, mediates the upregulation of RANKL expression in macrophages, thereby stimulating osteoclast formation (Aseel et al., 2019).

Th2 cells differentiate in response to stimulation by IL-2 and IL-4 and primarily secrete anti-inflammatory factors such as IL-4, IL-5, IL-9, IL-10, and IL-13, thereby inhibiting osteoclastogenesis (Raphael et al., 2015; Ren et al., 2017). Both *in vivo* and *in vitro* experiments (Amarasekara et al., 2018) have confirmed that IL-4 can inhibit bone resorption. IL-4 inactivates NF-κB and MAPK in OCs, inhibits NFATc1, and attenuates RANKL-induced osteoclastogenesis; simultaneously, IL-4 enhances OB production of OPG. IL-13 inhibits RANKL expression and the c-Fos gene via STAT1, thereby suppressing the proliferation and differentiation of OPCs. Furthermore, Th2 activation enhances the osteogenic effects of PTH, reduces the RANKL/OPG ratio, and significantly inhibits bone loss (Pacifici, 2010). Therefore, Th2 cells exhibit overall osteoprotective immunoregulatory effects.

### 3.2 B cells: from RANKL-producing effectors to regulatory protectors

B cells, as adaptive immune cells, develop from hematopoietic stem cell (HSC) precursors in the bone marrow. Their migration, homing, and maturation within the bone marrow are mediated by signaling via the chemokine C-X-C-motif ligand 12 (CXCL12). B cells participate in the process of bone remodeling in multiple ways. Research (Lagerquist et al., 2022) suggests that in transgenic mice lacking mature B cells, trabecular bone density increased despite reduced OPG levels; this may be related to reduced bone resorption resulting from a decrease in OC numbers. Numerous studies (Settem et al., 2021; Zeng et al., 2021) have shown that inducibly activated B cells produce large amounts of RANKL, promoting OC proliferation and differentiation. Concurrently, they produce inflammatory factors (such as TNF, TGF-β, and IFN-γ), which regulate B cell activation via negative feedback, further promoting OC-mediated bone resorption. However, mice with B-cell-specific RANKL knockout are partially resistant to the osteoporotic effects induced by ovariectomy (OVX) (Zhang, Z. et al., 2025). The bone-modulating effects of B cells are influenced by the microenvironment: under inflammatory conditions, B cells polarize toward an osteolytic phenotype and secrete G-CSF to promote OC proliferation (Frase et al., 2023); postmenopausal patients with OP exhibit reduced numbers of bone marrow B cells but enhanced G-CSF secretion, suggesting that estrogen deficiency abnormally affects B cell function via the CXCL12 pathway (Nordqvist et al., 2017; Ponte et al., 2020). The bone-modulating effects of B cells are also regulated by T cell subsets—B cells activated by Th1 factors can inhibit osteoclastogenesis, whereas those stimulated by Th2 factors promote it (Choi and Kim, 2003). Although studies on the effects of B cells on OBs are limited, evidence (Sun et al., 2018) suggests that B cells may inhibit osteoblast differentiation via CXCL3 and TNF signaling pathways.

Regulatory B cells (Bregs) are an important subset of B cells that play a role in bone protection by secreting anti-inflammatory mediators to inhibit osteoclastic activity (Sapra et al., 2025a). Among these, IL-35 promotes Breg differentiation via the STAT1/STAT3 pathway (Rosser and Mauri, 2015); IL-10 directly inhibits osteoclastogenesis by interfering with Ca^2+^ mobilization and NFATc1 signaling, and downregulates miR-7015-5p to promote osteogenic differentiation (Evans and Fox, 2007; Xiong et al., 2020); while TGF-β1 stimulates osteogenesis by upregulating runt-related transcription factor 2 (Runx2) via MAPK/Smad-mediated pathways, while simultaneously suppressing osteoclastogenic genes encoding tartrate-resistant acid phosphatase (TRAP) and cathepsin K (Lee et al., 2019). Consequently, in the TGF-β1-deficient osteoporotic microenvironment, the sustained expression of RANKL by OBs, combined with the attenuation of Runx2-mediated osteogenesis, collectively drives bone remodeling toward resorption.

### 3.3 Macrophages: phenotypic polarization as a determinant of skeletal fate

Macrophages and OCs share a common origin in the monocyte lineage. Macrophages (F4/80+)residing in bone tissue participate in the regulation of bone metabolism through phenotypicpolarization (Chen et al., 2025a). As key innate immune cells, macrophages do not exist in rigid binary states but rather adopt a continuum of functional phenotypes in response to local microenvironmental cues, ranging from pro-inflammatory, tissue-damaging to anti-inflammatory, tissue-reparative functions (Muñoz et al., 2020). While the historical nomenclature of classically activated (often termed “M1-like”) and alternatively activated (“M2-like”) macrophages, derived from *in vitro* polarization assays, provides a useful heuristic, it is now recognized that this simplification does not fully capture the complex, multidimensional, and often overlapping molecular states macrophages acquire *in vivo*.

Pro-inflammatory macrophages (often experimentally polarized to a state resembling the classical “M1-like” phenotype by stimuli such as LPS) exert pro-inflammatory effects and promote bone resorption, whereas reparative macrophages (often associated with an “M2-like” phenotype) are considered anti-inflammatory and support bone formation (Cheng et al., 2025). Studies (Chen K. et al., 2020) have shown that macrophages polarized towards a pro-inflammatory phenotype by lipopolysaccharide (LPS) produce pro-inflammatory cytokines such as IL-1β, IL-6, IL-12, TNF-α, and reactive oxygen species (ROS). These mediators directly or indirectly stimulate osteoclastogenesis and enhance bone resorption (Zhang B. et al., 2021).

IL-1, produced by myeloid cells such as macrophages, promotes OC migration and activation by increasing the expression of chemokines CCL19, CCL21, and their receptor CCR7 (Lee et al., 2017), and by activating NF-kB and AP-1 to promote RANKL-dependent osteoclastogenesis. In IL-1-deficient TNF transgenic mice, TNF-α-induced bone resorption depends to some extent on the presence of IL-1 (Zwerina et al., 2007). Furthermore, IL-1B promotes osteogenic differentiation of mouse BMSCs via the BMP/Smad pathway within a specific concentration range (Wang H. et al., 2020).

IL-6, produced by macrophages, acts on the IL-6 receptor to directly activate the JAK/STATand MAPK signaling pathways, thereby promoting the transcription of OC-related proteins and the release of inflammatory factors (Zhong et al., 2022). Studies (Zhang Y. et al., 2021) have shown that IL-6 can stimulate OBs to express RANKL and act synergistically with factors such as TNF-aand IL-1, thereby enhancing the differentiation and maturation of OPCs. In addition, IL-6 can upregulate sphingosine-1-phosphate receptor 2(S1PR2) on OPCs and facilitate their migration, playing an important role in systemic bone loss (Yu, 2021).

TNF-α, primarily produced by macrophages, acts on TNFR1 and TNFR2. By activating signaling pathways within OCs, it induces the release of various cytokines and chemokines, further promoting the inflammatory response (Zia et al., 2020). TNF-α cannot directly stimulate osteoclastogenesis but exerts its effects by upregulating RANKL: it directly stimulates cells such as OBs to express RANKL (Aseel et al., 2019); simultaneously, it increases the expression of Wnt/β-catenin pathway inhibitors such as DKK-1 or SOST, thereby disrupting OB function (Diarra et al., 2007). Furthermore, TNF-α can promote osteoclastogenesis synergistically with RANKL by activating the PI3K/AKT pathway via M-CSF. At specific concentrations, TNF-α can promote the early proliferation and differentiation of osteoprogenitor cells, suggesting dose-dependent and context-specific effects (Wang S. et al., 2025).

In contrast, macrophages with a reparative, anti-inflammatory phenotype (often referred to as “M2-like”), stimulated by Th2-secreted cytokines, secrete anti-inflammatory molecules (such as IL-4, IL-10, and IL-13) and osteogenic molecules (including BMP-2, TGFβ, and OPN), thereby exerting anti-inflammatory and immunomodulatory effects (Glass et al., 2014). These reparative macrophages can stimulate MSCs and OBs, thereby promoting bone mineralization. IL-4 inactivates NF-κB and MAPK in OCs, inhibits NFATc1, and attenuates RANKL-induced osteoclastogenesis; simultaneously, IL-4 enhances OB production of OPG (Amarasekara et al., 2018). IL-13 inhibits RANKL expression and the c-Fos gene via STAT1, thereby suppressing the proliferation and differentiation of OPCs. IL-10 directly inhibits osteoclastogenesis by downregulating RANKL and M-CSF expression (Wang Y. et al., 2020). Notably, the “M2-like” macrophage participates in the clearance of apoptotic cells, helping to maintain the homeostasis of bone remodeling.

These studies suggest that chronic inflammation driven by pro-inflammatory macrophages exacerbates the progression of OP. Conversely, by inhibiting OC function and bone resorption through anti-inflammatory cytokines (such as IL-4, IL-10, and IL-13), reparative macrophages have been established as key regulators of osteogenic activity. Therapeutic strategies that promote a shift in the macrophage balance from a pro-inflammatory toward a reparative phenotype may be an effective strategy for improving OP.

### 3.4 Neutrophils: double-edged swords in the bone remodeling arena

Neutrophils are generated from bone marrow precursors under the regulation of G-CSF and GM-CSF. They serve as the first line of defense in the innate immune system, clearing pathogens through phagocytosis, granule release, and the formation of neutrophil extracellular traps (NETs) (Liew and Kubes, 2019). In bone metabolism, the function of neutrophils is complex. Hajishengallis et al. (2016) propose that neutrophils can recruit Th17 cells and suppress B cells by secreting chemokines such as IL-8 and IL-17, thereby driving inflammatory bone loss. Recent studies (Ando et al., 2024; Cai et al., 2021) indicate that neutrophil senescence or dysfunction can directly or indirectly influence osteogenic and osteoclastic processes. In the early stages of bone healing, neutrophils secrete various pro-angiogenic growth factors (fibroblast growth factor (FGF)-2, platelet-derived growth factor (PDGF)) and osteogenic factors (TGF-β), exerting a protective effect on bone formation (Kovtun et al., 2018). Furthermore, *in vitro* co-culture experiments (Herath et al., 2018) have also demonstrated that neutrophils induce the expression of osteogenic markers (such as osteocalcin, alkaline phosphatase, and BMP) and mineralization. However, other studies have shown (Kim A. R. et al., 2020) that activated neutrophils secrete RANKL, generate ROS, and release pro-inflammatory cytokines that promote osteoclastogenesis, thereby contributing to osteoclastogenesis and maturation (Chakravarti et al., 2009; O’Neil et al., 2023). Notably, G-CSF-expanded neutrophils can induce apoptosis in MSCs and OBs via ROS (Bastian et al., 2018), while the osteogenic effect of neutrophil-MSC co-culture is influenced by IL-8 and TGF-β levels (Singh et al., 2012). Clearly, the osteogenic effect may depend largely on the activation state of neutrophils. In summary, the osteogenic or osteoclastic effects of neutrophils depend on their activation state and the local microenvironment, and the specific molecular mechanisms remain to be further elucidated.

### 3.5 Dendritic cells: antigen-presenting cells with bone-regulatory duality

Dendritic cells (DCs), primarily found in the bone marrow, are specialized antigen-presenting cells (APCs) that highly express major histocompatibility complex class II (MHC-II) molecules and co-stimulatory receptors (CD80/CD86). While initiating adaptive immune responses, they also exert significant bidirectional regulatory effects on bone metabolism. On the one hand, DCs produce cytokines such as IL-1, IL-6, and TNF-α, and influence the balance between bone resorption and bone formation through the Wnt/β-catenin and RANKL/OPG pathways (Chen, 2022). In addition, DCs can activate T cells (particularly by inducing Th17 polarization), trigger the secretion of IL-17 and TNF-α, and promote the production of RANKL, thereby stimulating osteoclast resorption activity (Wang B. et al., 2021). On the other hand, DCs secrete OPG, which antagonizes the phosphorylation of the NF-κB and MAPK pathways, inhibits RANKL-mediated osteoclastogenesis, reduces the expression of markers such as TRAP and cathepsin K, and alleviates bone loss (Cang et al., 2021). Moreover, IFN-β, primarily secreted by fibroblasts and dendritic cells in response to viral infection or inflammatory stimulation, exerts antiviral and immunomodulatory effects (Daman and Josefowicz, 2021). In addition to regulating osteoclastogenesis, IFN-β promotes OB proliferation and function, thereby facilitating bone formation (Liu X. et al., 2024). In inflammatory bone diseases such as rheumatoid arthritis, IFN-β is believed to have a protective effect by inhibiting osteoclastogenesis. Furthermore, Mature DCs can also promote the osteogenic differentiation of BMSCs through the transfer of miR-335, thereby enhancing bone defect repair (Cao et al., 2021). Other studies (Dou et al., 2018) have shown that graphene oxide-polyethyleneimine complexes loaded with miR-7b overexpression plasmids can inhibit the expression of a key fusion molecule in a dendritic cell-specific transmembrane protein, thereby significantly reducing OC fusion and resorption activity. In summary, DCs fine-tune the balance between resorption and formation by comprehensively regulating multiple signaling pathways.

### 3.6 Mast cells: estrogen-sensitive mediators of bone turnover

Mast cells (MCs) originate from the myeloid stem cell lineage in connective tissues throughout the body and can rapidly release large amounts of granules in response to inflammatory or allergic stimuli. Analysis of changes in serum biochemical markers of bone turnover suggests that MCs play a significant role in regulating bone turnover. First, MCs can release mediators such as histamine and platelet-activating factor to stimulate osteoblast proliferation, differentiation, and bone matrix deposition; among these, histamine exerts a significant stimulatory effect on OCs, and blocking H1 receptors can inhibit OVX-induced bone loss (Fischer et al., 2020). Several other MC mediators, including TNF-α, IL-6, and RANKL, also participate in the stimulation of OCs, whereas prostaglandin D2, substance P, IFN-γ, and IL-10 exert osteoprotective effects (Franco et al., 2023). Furthermore, MCs can inhibit OC activity and the process of bone resorption by releasing TGF-β, IL-4, and MC-derived growth factors (Yokota et al., 2021). Estrogen deficiency is a key trigger for MC-mediated bone pathology: the number of bone marrow MCs is significantly increased in OVX rats and postmenopausal patients, whereas the osteoclastic potential of MCs is suppressed in the presence of estrogen (Lampiasi, 2022; Lesclous and Saffar, 1999). Thus, MCs exert bidirectional regulation on bone metabolism, and estrogen deficiency acts as a key molecular switch that unlocks their osteoclastic activity.

### 3.7 Natural killer cells: innate lymphocytes with context-dependent osteoactivity

Natural killer (NK) cells traditionally originate from hematopoietic stem cells (HSCs) in the bone marrow and regulate the function of other immune cells by modulating the local microenvironment (Scoville et al., 2017). It has now been demonstrated that they possess the ability to mature within secondary lymphoid tissues (SLTs), where they acquire adaptive characteristics, including memory function (Scoville et al., 2017). In addition to their classic tumor-lytic activity, NK cells can eliminate virus-infected and stress-affected cells through balanced signaling between activating and inhibitory receptors (Joshua and Yiping, 2011; Parham, 2005; Trinchieri, 1989). These lymphocytes also reshape the local microenvironment by mediating antibody-dependent cellular cytotoxicity (ADCC) and secreting pro-inflammatory mediators (such as IFN-γ and chemokines) that regulate neighboring immune cells (Fauriat et al., 2010).

It is worth noting that cytotoxic NK cells can stimulate the production of TNF-α, leading to its accumulation in patients with rheumatoid arthritis (RA), and by secreting IL-22, they cause excessive proliferation of fibroblast-like synoviocytes, leading to synovial hyperplasia and bone destruction (Kucuksezer et al., 2021). In a mouse model of collagen-induced arthritis, NK cells express RANKL and stimulate OC formation, whereas depletion of NK cells inhibits bone resorption. IL-15-activated NK cells can promote bone resorption by upregulating leukocyte function-associated antigen-1 (LFA-1) and DNAX auxiliary molecule-1 (DNAX auxiliary molecule-1, DNAX-1), which act as ligands for the receptor adhesion molecules 1 and CD155 on the surface of OCs, thereby inhibiting OC development and function and, consequently, bone resorption (Saxena et al., 2021). Therefore, NK cells can both express TNF-α and RANKL to promote osteoclastogenesis and mediate OC inhibition via adhesion molecules; their net effect is highly dependent on the tissue microenvironment, and the specific regulatory mechanisms require further investigation.

## 4 GM–immune–bone axis in OP

### 4.1 GM composition in OP: characteristics and shifts

The GM is a complex microecosystem colonizing the gut, often referred to as the “second genome.” It is primarily composed of the *Bacteroidetes*, *Firmicutes*, *Actinobacteria*, and *Proteobacteria phyla*, with *Bacteroidetes* and *Firmicutes* accounting for approximately 90% of the total (Rinninella et al., 2019; Spencer et al., 2019). *Bacteroidetes* are involved in food digestion and metabolism; *Firmicutes* are closely associated with the production of certain key metabolic products (SCFAs); and *Proteobacteria* and *Actinobacteria* are significantly linked to the host’s immune response and inflammatory status (Waldbaum et al., 2023). In summary, the GM exerts a profound influence on host health through pathways such as nutrient metabolism, vitamin synthesis, maintenance of the intestinal barrier, and immune regulation.

Numerous studies have shown that alterations in GM diversity/abundance and specific metabolites are closely associated with the development of OP. Clinical studies (Li et al., 2019) have demonstrated that individuals with reduced bone mass exhibit increased abundance of the *Bacteroidetes phylum* and decreased abundance of the *Firmicutes phylum* in their feces; furthermore, *Bacteroidetes* abundance is negatively correlated with bone density, while *Firmicutes* abundance is positively correlated with it (Lan et al., 2021). Similarly, Castaneda et al. (2021) observed significant changes in the GM community structure of aged mice treated with antibiotics in an animal model. These changes not only affected the strength of the mice’s bone tissue but also led to a decrease in osteocalcin levels. Zhang et al. (2024) found in a further study that supplementation with *Bifidobacterium* can suppress the expression of intestinal inflammatory cytokines such as TNF-α and IL-6, thereby alleviating intestinal inflammation and inhibiting osteoclastogenesis, which in turn increases bone density. Furthermore, compared to conventional mice, germ-free (GF) mice exhibited reduced OC numbers and increased bone mass; however, their bone mass returned to normal levels 4 weeks after receiving a normal GM transplant, directly confirming the regulatory role of GM in bone homeostasis (Sjögren et al., 2012).

Several other beneficial bacteria have also demonstrated bone-protective potential (Table 4). Takimoto et al. (2018) reported that oral administration of *Bacillus subtilis C-3102* increased bone density in postmenopausal women and reduced serum levels of the tartrate-resistant acid phosphatase (TRAP). Zhang et al. (2015) found that *Lactobacillus reuteri* exerts a significant positive regulatory effect on bone tissue in mice with type I diabetes. This strain enhances OB activity by promoting the expression of the Wnt10b signaling pathway, effectively slowing bone loss. Furthermore, *Lactobacillus* can reduce systemic inflammation, improve the intestinal barrier, and, by inhibiting B-cell production of RANKL, jointly suppress osteoclastogenesis in the presence of M-CSF (Hamedi-Kalajahi et al., 2010).

**TABLE 4:** Summary of the major functions of GM in the gut-immune-bone axis.

Gut microbiota species	Phylum	Major functions in the gut-immune-bone axis	Effects on bone metabolism	References
*Bifidobacterium* spp.	*Actinobacteria*	Promotes Treg cell differentiation; inhibits inflammatory cytokines (TNF-α, IL-6); upregulates OPG and Wnt10b/β-catenin pathway; enhances gut barrier function	Bone-protective (↑ bone mass, ↓ osteoclastogenesis)	Chen et al. (2025b)
*Lactobacillus* spp.	*Firmicutes*	Promotes SCFA production; regulates Th17/Treg immune homeostasis; improves calcium absorption; produces metabolites (e.g., indicaxanthin, chlorogenic acid); corrects dysbiosis; *L. reuteri* secretes antimicrobial peptides via NOD2 and hormones (serotonin, GIP, PYY, etc.); *L. acidophilus* and *L. rhamnosus GG* enhance gut barrier and modulate immunity	Bone-protective (↑ bone density, ↓ bone resorption/osteoclastogenesis, ↑ bone formation)	Guo et al. (2023)
*Bacillus* spp.	*Firmicutes*	Balances Treg/Th17 ratio (*B. clausii*); decreases bone resorption markers (TRAP); induces SCFA production to enhance Breg function	Bone-protective (↑ BMD, ↓ bone resorption)	Dar et al. (2018)
*Butyricicoccus pullicaecorum*	*Firmicutes*	Regulates Th17/Treg immune homeostasis	Bone-protective (anti-inflammatory, potent anti-osteoporotic)	Zhu et al. (2024)
*Faecalibacterium prausnitzii*	*Firmicutes*	Suppresses osteogenic differentiation via butyrate production	Potentially detrimental to bone formation under certain contexts	Moon et al. (2023)
*Clostridium* spp.	*Firmicutes*	Bregs to increase IL-10/Treg proportion; key producer of secondary BAs	Bone-protective (modulates immunity to favor bone health)	Atarashi et al. (2011)
Prevotellaceae spp.	*Bacteroidetes*	Drives Th17 differentiation (e.g., *P. copri*); positive correlation with systemic inflammation and RA onset; *P. histicola* restores bone mass by improving gut permeability	Context-dependent: Pro-inflammatory/osteoporotic (most *Prevotella*); bone-protective (P. histicola)	Jie et al. (2025)
*Bacteroides* spp.	*Bacteroidetes*	*B. fragilis* induces anti-inflammatory factors (TGF-β, IL-10) in DCs/Tregs and drives Breg IL-10 secretion; its toxin may recruit Th17 cells via STAT3; other *Bacteroides spp*. positively correlate with glucose/lipid metabolism and bone density (*Parabacteroides*)	Context-dependent: mainly bone-protective via immune regulation; some pro-inflammatory roles	Zheng, C. et al. (2025)
*Parabacteroides* spp.	*Bacteroidetes*	Positive regulation in glucose and lipid metabolism; strong positive correlations with trabecular BMD (Tb.BMD) and trabecular number (Tb.N); increased abundance in PMO patients	Bone-protective (metabolic benefits)	Becerra-Cervera et al. (2025)
*Muribaculum*	*Bacteroidetes*	Regulates T cells; strong negative correlations with intestinal inflammatory factors and bone resorption factors; strong positive correlations with intestinal barrier proteins and bone formation factors	Bone-protective (anti-inflammatory, promotes bone formation)	Roberts et al. (2023)
*Fusobacterium nucleatum*	*Fusobacteria*	Promotes polarization toward a pro-inflammatory (“M1-like”) macrophage phenotype via AKT2 signaling exacerbates inflammation, pro-inflammatory cytokine expression, and monocyte activation	Pro-resorptive (↑ colonic and systemic inflammation, ↑ bone loss)	Liu et al. (2019)
*Segmented Filamentous Bacteria*	*Bacillota*	Promotes Th17 differentiation via serum amyloid A (SAA) production; drives intestinal TNF^+^ T cell and Th17 expansion, leading to bone loss	Pro-resorptive (↑ Th17-mediated osteoclastogenesis)	Hathaway-Schrader et al. (2022)

Conversely, the overgrowth of certain pathogens or opportunistic pathogens drives bone loss. Overproliferation of *Klebsiella* can induce chronic intestinal inflammation, promote the release of TNF-α and IL-6, enhance OC activity, and disrupt the intestinal barrier (Liang et al., 2023; Sun et al., 2024). The proliferation of *Escherichia* and *Shigella* not only exacerbates intestinal inflammation but also interferes with the absorption of vitamin D and calcium; their metabolic byproducts, such as propionate, can also disrupt the osteogenic-resorptive balance (Sun et al., 2024).

### 4.2 GM metabolites: regulation of immune cells and bone homeostasis

The GM and its vast repertoire of metabolites constitute an integrated signaling network that profoundly shapes the systemic immune landscape. Rather than functioning as independent entities, the microbial community and its biochemical output act in concert to regulate the differentiation, polarization, and effector functions of T cells, B cells, and macrophages, thereby dictating the balance between bone resorption and formation (Zhang et al., 2023) (Figure 2).

**FIGURE 2:**
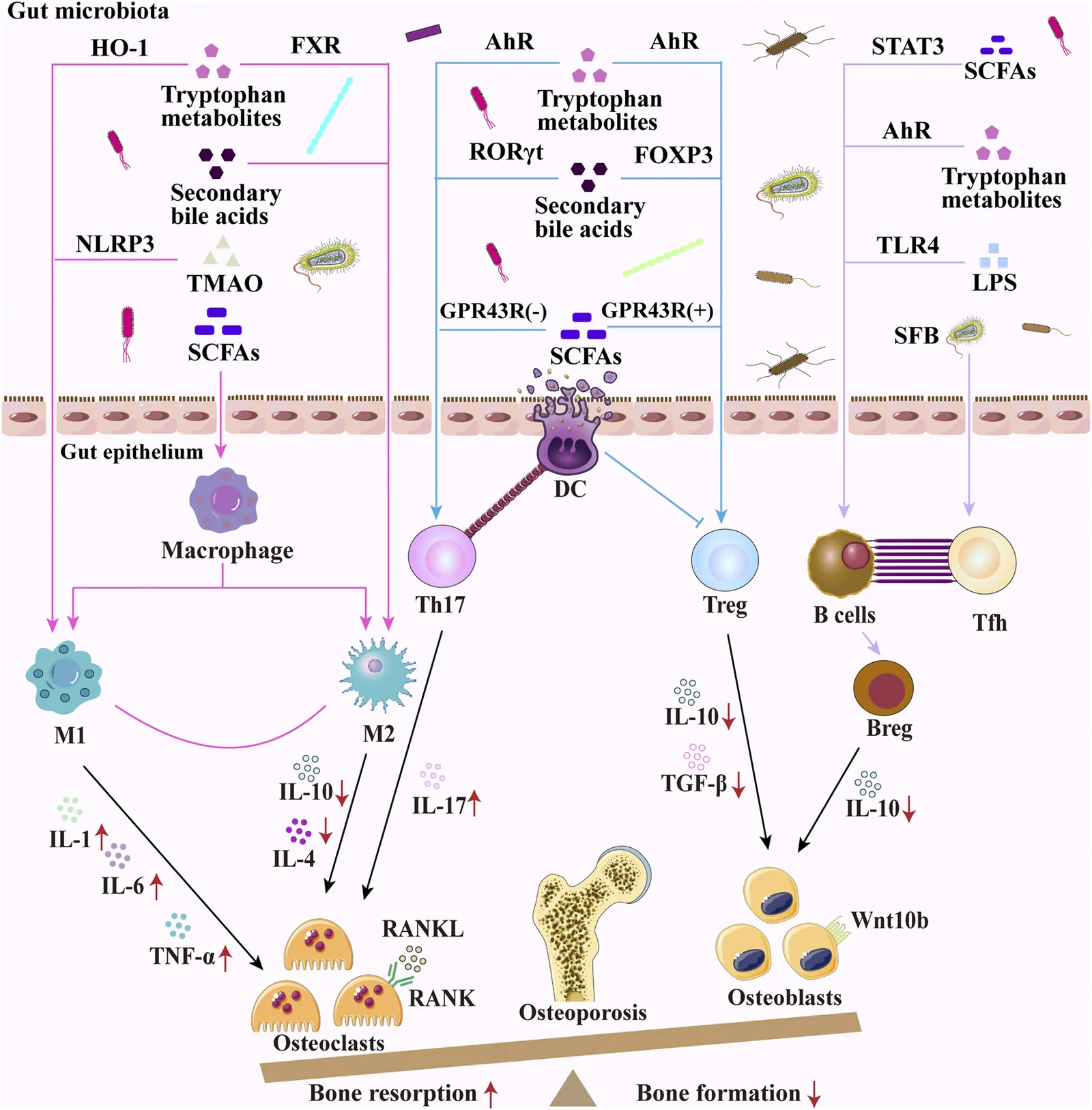
GM Metabolites regulate bone metabolism via immune-bone axis. GM-derived metabolites, including SCFAs, tryptophan metabolites, secondary BAs, LPS, and TMAO, act on intestinal epithelial cells and immune cells such as dendritic cells, macrophages, Treg/Th17 cells, B cells, and Bregs. Through signaling pathways including AhR, FXR, TLR4, NLRP3, and GPR43, these metabolites modulate the expression of pro-inflammatory and anti-inflammatory factors, thereby regulating osteoblastic bone formation and osteoclastic bone resorption in OP.

#### 4.2.1 SCFAs: central regulators of the Treg/Th17 rheostat

SCFAs—primarily acetate, propionate, and butyrate—are generated by fermentation of dietary fiber by *Firmicutes* (e.g., *Faecalibacterium*, *Roseburia*, *Clostridium clusters IV/XIVa*) and *Bacteroidetes* (Zhang et al., 2023). These metabolites exert profound immunomodulatory effects through histone deacetylase (HDAC) inhibition and activation of G-protein-coupled receptors (GPR41, GPR43, GPR109A), directly influencing T-cell fate and macrophage polarization (Behler-Janbeck et al., 2024; Heberling et al., 2013).

The GM composition directly dictates the SCFA pool and thus the Treg/Th17 equilibrium. Multiple probiotic strains have been shown to restore the Th17/Treg balance through SCFA-dependent pathways. For instance, *Lactobacillus rhamnosus GG* reduces IL-17^+^ Th17 cells and increases CD25^+^Foxp3^+^ Treg cells by activating TLR2, an effect that is amplified by butyrate production (Jia et al., 2020). *Bacillus* clausii corrects the Treg/Th17 imbalance in OVX mice, reducing pro-inflammatory IL-6, IL-17, and TNF-α while elevating anti-inflammatory IL-10 and IFN-γ, an effect linked to increased SCFA availability (Dar et al., 2018). *Lactobacillus casei CCFM1074* upregulates the Treg/Th17 ratio via enhanced butyrate production, ameliorating arthritis-associated bone loss (Fan et al., 2021). Conversely, dysbiotic expansion of Segmented filamentous bacteria (SFB) or *Prevotella* species promotes Th17 differentiation through STAT3-dependent pathways, driving osteoclastogenic IL-17 and RANKL expression (Calcinotto et al., 2018; Chen B. et al., 2020; Durant et al., 2010). *Bacteroides fragilis* toxin can also recruit Th17 cells via STAT3 activation (Durant et al., 2010; Wu et al., 2009). Thus, the SCFA–Treg/Th17 axis serves as a critical checkpoint where GM composition and metabolic output converge to determine skeletal fate. Notably, various bacteria, including *Clostridium*, *Bacteroides*, *Bifidobacterium*, and *Lactobacillus* can promote Treg development, while Bregs induced by *Clostridium* stimulation further increase Treg proportions and IL-10 expression, reducing Th17 cells and IL-17 levels (Hsu and Pacifici, 2018; Qi et al., 2024).

Beyond their immunomodulatory actions through Treg/Th17 polarization, SCFAs exert direct, cell-autonomous effects on OCs and OBs that are independent of T-cell intermediaries. Propionate and butyrate directly suppress osteoclast differentiation and bone resorption by inducing metabolic reprogramming of OPCs: they enhance glycolysis at the expense of oxidative phosphorylation, thereby downregulating the expression of essential osteoclast genes, including TRAF6 and NFATc1. This metabolic shift renders OPCs energetically incompatible with the high demands of differentiation and fusion. Concurrently, SCFAs inhibit RANKL-induced NFATc1 nuclear translocation and c-Fos expression through GPR43-mediated signaling and histone deacetylase (HDAC) inhibition—a dual mechanism that suppresses OC-specific gene transcription (TRAP, cathepsin K, DC-STAMP) while preserving bone formation (Lucas et al., 2018). Importantly, butyrate acts as a non-redundant permissive signal for parathyroid hormone (PTH)-induced bone anabolism: butyrate is required for PTH to expand bone marrow Tregs via GPR43 signaling in dendritic cells and GPR43-independent pathways in T cells; these Tregs then stimulate CD8^+^ T cells to produce the osteogenic Wnt ligand Wnt10b, which activates canonical Wnt/β-catenin-dependent bone formation (Li et al., 2020). GM depletion abolishes PTH-induced bone formation, an effect fully rescued by butyrate repletion. These findings establish SCFAs not merely as immune rheostats but as direct metabolic checkpoints that couple gut microbial fermentation to OC energy metabolism and OB-activating Wnt signaling.

#### 4.2.2 Bile acids: dual regulation of Th17, Treg, and Breg cells via FXR and TGR5

Bile acids (BAs) are cholesterol derivatives metabolised by GM (e.g., *Clostridium, Bacteroides, Bifidobacterium*) into secondary forms such as lithocholic acid (LCA), deoxycholic acid (DCA), and ursodeoxycholic acid (UDCA) (Cai et al., 2022). These metabolites signal through the farnesoid X receptor (FXR) and the G-protein-coupled receptor 5 (TGR5), which are widely expressed on immune cells and bone marrow stromal cells (Boufker et al., 2011; Wang L. et al., 2025; Wang et al., 2018).

BA–FXR signaling promotes osteoblast differentiation via Wnt/β-catenin and ERK pathways, while suppressing RANKL-induced osteoclastogenesis through inhibition of NFATc1 and c-Fos. TGR5 activation by secondary BAs (e.g., LCA, DCA) induces macrophage polarization toward a reparative (“M2-like”) phenotype, enhances IL-10 secretion, and inhibits NLRP3 inflammasome assembly via the cAMP–PKA pathway, thereby limiting pro-inflammatory cytokine release (Biagioli et al., 2017; Pols et al., 2011; Zhao C. et al., 2023). Additionally, BAs modulate adaptive immunity: 3-oxoLCA directly binds RORγt to inhibit Th17 differentiation, whereas other BA species promote Foxp3^+^ Treg generation through FXR-dependent DC modulation (Campbell et al., 2020; Hang et al., 2019). Homeostasis of RORγt^+^ Tregs depends on the overall intestinal BA pool rather than individual secondary BAs (Song et al., 2020).

The GM not only produces these BA metabolites but also directly influences B-cell immunity. *Bacteroides fragilis*, via its capsular polysaccharide A, induces regulatory B cell (Breg) differentiation and IL-10 secretion, which suppresses osteoclastogenesis by inhibiting JAK/STAT signaling in OPCs (Dawalibi et al., 2024; Ramakrishna et al., 2019). *Bifidobacterium longum FSHHK13M1* upregulates VDR, OPG, and Wnt10b/β-catenin pathways, increases Breg proportions and IL-10/IFN-γ levels, while reducing TNF-α, IL-6, and IL-17 (Hao et al., 2025). Conversely, opportunistic pathogens such as *Escherichia coli* bind TLR4 on B cells to activate MyD88/NF-κB, exacerbating OC-mediated resorption—an effect blocked by probiotics that preserve intestinal barrier integrity and upregulate tight junction proteins (Wei et al., 2021). Thus, the BA–Breg–OPG axis represents a key osteoprotective circuit, where microbial BA metabolism and direct bacterial–B-cell crosstalk jointly fine-tune bone homeostasis. Restoration of BA balance, for instance via *Lactobacillus reuteri* promoting secondary BA production, significantly increases serum 25-hydroxy-vitamin levels and reduces fracture risk (Jones et al., 2013; Qiao et al., 2022).

#### 4.2.3 LPS: inflammatory relay from barrier disruption to pro-inflammatory (“M1-like”) macrophage polarization

LPS, a major component of Gram-negative bacterial outer membranes (e.g., *E. coli, Klebsiella pneumoniae, Shigella*), acts as a potent endotoxin that drives bone resorption via TLR4-mediated signaling (Horton et al., 2014; Wen et al., 2023). When gut dysbiosis increases intestinal permeability, LPS translocates into the circulation, activating the MyD88/NF-κB and TRIF/IRF-3 cascades on macrophages (Płóciennikowska et al., 2015; Raetz and Whitfield, 2002). This signaling promotes a pro-inflammatory macrophage phenotype, leading to robust secretion of IL-1β, IL-6, TNF-α, and ROS—cytokines that directly stimulate osteoclastogenesis and suppress OB function (Pajarinen et al., 2019; Shapouri-Moghaddam et al., 2018; Wang and He, 2020). LPS also upregulates MHC-II and co-stimulatory molecules on dendritic cells and recruits neutrophils via CXCL1/2 (Aries et al., 2024; Gröbner et al., 2014). LPS synergizes with RANKL to enhance OC differentiation, and chronic LPS exposure is a major risk factor for OP (AlQranei et al., 2021; Kwon et al., 2021).

The GM composition critically determines the LPS load and its immune consequences. Overgrowth of *Klebsiella* and *Escherichia–Shigella* drives chronic intestinal inflammation and enhances polarization of macrophages toward a pro-inflammatory (“M1-like”) phenotype, increasing TNF-α and IL-6 release, which in turn upregulate RANKL and disrupt the intestinal barrier (Liang et al., 2023; Sun et al., 2024; Xu et al., 2023). In contrast, probiotics such as *Lactobacillus acidophilus* and *Bifidobacterium longum* promote macrophage polarization toward the anti-inflammatory reparative phenotype (“M2-like”) (Hao et al., 2025; Kim et al., 2022). An extracellular protein from *L. acidophilus* enhances macrophage phagocytosis and reduces LPS-induced RANKL expression, facilitating the transition to reparative macrophages (“M2-like”) (Pugazhendhi et al., 2024). EPS624, a novel extracellular protein from *Bifidobacterium longum*, induces intestinal tolerance via TLR2 and establishes an anti-inflammatory microenvironment through IL-10 and Foxp3 expression (Wallimann et al., 2021). Notably, the synthetic TLR2 ligand Pam3CSK4 can upregulate RANK in bone marrow cells, promoting osteoclastogenesis. *Akkermansia muciniphila*-derived extracellular vesicles reduce NO, TNF-α, and IL-1β production in macrophages, attenuating inflammatory responses and exerting osteoprotective effects (Zheng et al., 2023). Restoring the balance between pro-inflammatory and reparative macrophage phenotypes by modulating the GM—either by suppressing LPS-producing pathobionts or supplementing with probiotics that promote a reparative (“M2-like”) macrophage phenotype—is therefore a promising therapeutic strategy for OP.

#### 4.2.4 Tryptophan metabolites: AhR-mediated immune tolerance and osteogenic reprogramming

Tryptophan is metabolised by GMs (e.g., *Bifidobacterium, Clostridium, Lactobacillus*) into indole derivatives such as indole-3-propionic acid (IPA) and indole-3-acetic acid (IAA), as well as kynurenine pathway products (Åkesson et al., 2018; Wu et al., 2022). These metabolites activate the aryl hydrocarbon receptor (AhR), a transcription factor highly expressed in Th17 cells, Tregs, and DCs, thereby orchestrating immune–bone crosstalk (Rahul and Tracy, 2018).

AhR activation exerts context-dependent effects: in Th17 cells, it promotes IL-22 production and enhances pathogenicity, while in the presence of certain indole metabolites, it drives Foxp3^+^ Treg differentiation via dendritic cell modulation (Hauben et al., 2008; Sun et al., 2020; Yeste et al., 2014). IPA and IAA have emerged as potent osteoprotective molecules; they activate intestinal AhR and stimulate Wnt/β-catenin signaling, mitigating TNF-α- and IFN-γ-induced barrier dysfunction, while promoting IL-10 secretion from reparative macrophages that diffuses to the bone marrow to inhibit osteoclastogenesis and support osteoblast formation (Chen C. et al., 2024). IPA also dose-dependently suppresses pro-inflammatory cytokines via the TLR4/MyD88/NF-κB and TRIF/NF-κB pathways (Wang X. et al., 2025).

The GM directly influences this axis through specific taxa. *Bifidobacterium* and *Clostridium* species are major producers of IPA and IAA, and their abundance correlates positively with bone mass. Supplementation with *Lactobacillus* or *Bifidobacterium* increases circulating indole metabolites, which in turn upregulate intestinal AhR activity and restore the Treg/Th17 balance (Chen Y. et al., 2024). Notably, the tryptophan metabolite melatonin, produced by the GM, increases SCFA production and reduces TMAO, while restoring the balance between pro-inflammatory and reparative macrophages and lowering pro-inflammatory factors, thereby alleviating OP-related symptoms (Chen Y. et al., 2024). Thus, the tryptophan–AhR–Breg/M2 axis provides a direct mechanistic link from microbial metabolism to osteoimmune homeostasis.

#### 4.2.5 Trimethylamine N-Oxide (TMAO): a pro-osteoporotic metabolite via NLRP3 inflammasome activation

TMAO is a GM metabolite derived from dietary choline and carnitine, primarily produced by *Firmicutes* and *Proteobacteria*. Elevated plasma TMAO correlates with reduced BMD and promotes osteoclastogenesis through activation of the NLRP3 inflammasome and NF-κB signaling. TMAO induces excessive ROS, triggering NLRP3-mediated IL-1β and IL-18 release, which enhances RANKL-induced osteoclast differentiation. TMAO also inhibits osteogenic differentiation of BMSCs while promoting adipogenic differentiation via PPARγ upregulation, and increases pro-inflammatory cytokines (IL-1β, TNF-α, IL-6) (Lin et al., 2020). NLRP3 deficiency significantly promotes osteoblast differentiation and accelerates bone healing (Zhao W. et al., 2023). Reducing TMAO-producing microbial taxa or inhibiting their metabolic pathways is emerging as a novel intervention target. Probiotics such as *Lactobacillus* and *Bifidobacterium* have been shown to lower TMAO levels, thereby attenuating its deleterious effects on bone.

### 4.3 Hormonal regulation of the GM–immune–bone axis: estrogen and PTH

#### 4.3.1 Estrogen deficiency: driving dysbiosis and inflammatory bone loss

Declining estrogen levels not only trigger menopausal syndrome but also maintain a close bidirectional regulatory relationship with GM dysbiosis. In postmenopausal women (Guan et al., 2023), integration of 16S rRNA gene sequencing with liquid chromatography–mass spectrometry (LC-MS) metabolomics analysis revealed a decrease in both bacterial richness and diversity; furthermore, significant differences in GM abundance were observed at the phylum and genus levels. Specifically, compared with healthy controls, patients with postmenopausal osteoporosis (PMO) exhibited increased abundances of *Parabacteroides, Lactobacillus,* and *Bacteroides* in the gut, while the abundances of *Blautia, Clostridium, Lachnospiraceae_UCG-001*, *Lachnospiraceae_UCG-004*, and *Prevotella* were reduced. Similar results were observed in an OVX rat model (Chen et al., 2025c), which not only mimics the hormonal state of postmenopausal humans but also exhibits characteristic changes in GM similar to those in humans. Notably, treatment with *Prevotella histicola* partially restored bone mass in OVX mice. This effect may be mediated by the upregulation of tight junction proteins (such as claudin and occludin-1) to maintain intestinal barrier integrity, whose deficiency leads to increased intestinal permeability, triggering endotoxin translocation and chronic low-grade inflammation, which in turn promotes Th17 cell expansion and the expression of TNF-α, RANKL, and IL-17, thereby inducing bone loss (Li et al., 2016; Sapir-Koren and Livshits, 2017; Wang et al., 2021b; Xu et al., 2017). Other studies (Khan et al., 2010) have also found that estrogen can alter the structure of the GM by regulating TLR expression in the intestine, thereby influencing the activation of the immune system. Supplementation with *Prevotella histicola* partially restores bone mass in OVX mice; probiotics such as *Lactobacillus reuteri* and *Bifidobacterium lactis* can inhibit activation of pro-inflammatory (“M1-like”) macrophages, alleviate intestinal inflammation, and prevent bone loss (Kau et al., 2011; Lee et al., 2019; Martinmillan et al., 2010).

#### 4.3.2 PTH: microbial dependence and dual bone effects

PTH is the primary endocrine regulator of extracellular calcium and phosphate levels and exerts a bidirectional regulatory effect on bone metabolism—that is, it promotes bone formation and inhibits bone resorption. Continuous exposure to parathyroid hormone (cPTH) stimulates bone resorption, leading to bone mass loss; conversely, intermittent exposure to parathyroid hormone (iPTH) stimulates OBs, thereby promoting bone formation. Studies (Campos et al., 2020) have shown that iPTH can prevent bone loss in OVX rats. Similarly, in studies (Tripto-Shkolnik et al., 2024) of postmenopausal women, PTH exerted a significant osteogenic effect and effectively prevented bone loss. Yu et al. (2020) reported that, compared to mice treated with antibiotics, cPTH increased trabecular bone volume fraction and trabecular bone thickness in untreated mice. Furthermore, iPTH treatment did not induce increased bone mass in either knockout mice or antibiotic-treated wild-type mice (Li et al., 2020). Based on these results, it is hypothesized that GMs play a key role in mediating PTH-level regulation of bone metabolism. In fact, studies (Yu et al., 2020) have found that intestinal SFB mediate PTH-induced proliferation of intestinal TNF^+^T cells and Th17 cells, and increase their sphingosine-1-phosphate (S1P)-receptor 1-mediated efflux from the intestine and recruitment to the bone marrow, leading to bone loss. In addition to the GM itself, its metabolites also participate in PTH-induced bone metabolism. A study by Li et al. (2020) confirmed that butyrate, a metabolite produced by the GM, is essential for PTH-mediated bone metabolism. Butyrate increases the number of Treg cells, which stimulate CD8^+^ T cells in the bone marrow to produce the osteogenic Wnt ligand (Wnt10b), activating the Wnt-dependent bone-forming GM, thereby stimulating bone formation and increasing bone mass. At the same time, iPTH requires the GM to increase the production of TGF-β1 and insulin-like growth factor-1 (IGF-1) in bone marrow. In GF female mice in which the GM was eradicated with broad-spectrum antibiotics, iPTH failed to induce bone formation. A recent study (Lawenius et al., 2020) indicates that *Akkermansia muciniphila* can elevate serum PTH levels in normal mice, but this effect is absent in OVX mice. However, in postmenopausal women, a multi-strain probiotic supplement reduced levels of alkaline phosphatase and C-terminal cross-linked collagen type I, as well as PTH levels, compared to a placebo group (Jafarnejad et al., 2017). Therefore, identifying key bacterial strains capable of regulating PTH levels is crucial for determining potential targets for the effective prevention and treatment of OP.

#### 4.3.3 Estrogen and PTH: comparative signaling, immune modulation, and cross-regulatory networks with the GM

Estrogen and PTH represent two principal endocrine regulators of bone metabolism, yet they operate through fundamentally distinct receptors, signaling cascades, and temporal dynamics. Understanding their similarities and differences, as well as their reciprocal interactions with the GM and immune system, is essential for deciphering the complex hormonal circuitry within the gut-immune-bone axis.

##### 4.3.3.1 Distinct signaling pathways and bone outcomes

Estrogen acts predominantly via estrogen receptors α and β (ERα/ERβ) on OBs, OCs, and immune cells. Classical genomic signaling involves ER translocation to the nucleus and regulation of target genes (e.g., OPG, RANKL, Foxp3), while rapid non-genomic effects occur through membrane-associated ERs and G protein-coupled estrogen receptor 1 (GPER1), modulating MAPK/ERK and PI3K/AKT pathways. Estrogen deficiency, as in menopause, relieves suppression of RANKL expression in OBs and T cells, expands the Th17 pool, reduces Treg numbers, and shifts macrophage polarization toward the pro-inflammatory M1 phenotype—collectively driving bone resorption.

By contrast, PTH signals through the PTH/PTHrP receptor (PPR), a G-protein-coupled receptor that activates both Gs (cAMP/PKA) and Gq (PLC/PKC) pathways. The biological outcome is strictly schedule-dependent: continuous PTH exposure promotes bone catabolism, whereas iPTH exerts robust anabolic effects by increasing OB number and activity. Immunologically, iPTH expands bone marrow Tregs via cAMP-PKA-CREB signaling, which in turn stimulates CD8^+^ T cells to produce Wnt10b, a potent osteogenic Wnt ligand. Simultaneously, iPTH suppresses Th17 differentiation and reduces systemic TNF-α and IL-17 levels (Yu et al., 2020).

##### 4.3.3.2 Shared and divergent immunomodulatory features

Both hormones converge on the Treg/Th17 rheostat but with opposite net effects under pathological conditions. Estrogen maintains a high Treg/Th17 ratio under physiological conditions, while its withdrawal skews the balance toward Th17 predominance. PTH, especially in its intermittent form, also promotes Treg expansion, suggesting that both hormones support a bone-protective immune milieu. However, the underlying molecular mechanisms differ: estrogen directly upregulates Foxp3 through ER binding, whereas PTH relies on a GM-butyrate intermediary to amplify Treg numbers.

Another divergence lies in macrophage regulation. Estrogen inhibits polarization toward a pro-inflammatory (“M1-like”) macrophage phenotype and promotes reparative (“M2-like”) macrophage phenotypes through ERα-dependent suppression of NF-κB and NLRP3 inflammasome activation (Toniolo et al., 2015). PTH, in contrast, has a more modest and indirect effect on macrophages, primarily through modulation of T-cell-derived cytokines and IGF-1 production in the bone marrow microenvironment.

##### 4.3.3.3 The GM as a context-dependent mediator

A striking distinction between the two hormonal pathways is their differential dependence on the GM. The bone-anabolic action of iPTH is completely abrogated in GF mice or following broad-spectrum antibiotic treatment, underscoring an absolute requirement for microbial metabolites—particularly butyrate. Butyrate enhances Treg differentiation, stimulates CD8^+^ T-cell Wnt10b production, and is indispensable for iPTH-induced bone formation (Li et al., 2020). In contrast, estrogen deficiency-associated bone loss is GM-dependent but not entirely ablated by GF status; rather, dysbiosis amplifies the inflammatory cascade. Ovariectomy reduces microbial diversity, depletes butyrate-producing genera (*Clostridium, Lachnospiraceae*), and increases gut permeability, facilitating LPS translocation and systemic inflammation (Jin et al., 2026). However, estrogen supplementation can partially restore microbial composition and barrier integrity, indicating a bidirectional regulatory loop.

##### 4.3.3.4 Cross-regulatory networks and interplay

Emerging evidence suggests reciprocal modulation between estrogen and PTH pathways. Estrogen can modulate PTH secretion indirectly through calcium homeostasis: estrogen enhances intestinal calcium absorption by upregulating TRPV6 and calbindin-D9k, thereby lowering PTH secretion (Dong et al., 2014). At the immune level, both hormones intersect at the level of RANKL/OPG regulation—estrogen directly suppresses RANKL and upregulates OPG in OBs, while PTH increases RANKL transiently but also promotes OPG through Treg-mediated Wnt10b signaling, creating a temporally coordinated balance.

The GM serves as a common node that integrates these hormonal signals. Estrogen deficiency and iPTH both alter the abundance of *Lactobacillus*, *Bifidobacterium*, and *Akkermansia*, though in opposite directions. GM-derived metabolites (SCFAs, secondary BAs, tryptophan derivatives) can modulate both ER and PPR sensitivity, forming a feed-forward regulatory loop. For instance, secondary BAs (e.g., LCA, DCA) activate TGR5 on enteroendocrine L-cells to stimulate GLP-1 secretion (Trabelsi et al., 2015), which not only enhances osteoblast proliferation but also potentiates insulin signaling that cross-talks with PTH anabolic effects.

In summary, while estrogen and PTH utilize distinct receptors, cellular targets, and temporal patterns, they converge on shared immune checkpoints—Treg/Th17 balance, macrophage polarization, and RANKL/OPG ratio—and are both intimately connected to the GM. The GM acts both as a mediator (for PTH, an absolute requirement for butyrate) and as a modulator (for estrogen, amplifying inflammatory bone loss through dysbiosis). Therapeutic strategies that concurrently target hormonal signaling and microbial ecology may offer synergistic benefits for OP management, particularly in postmenopausal women with impaired PTH responsiveness.

### 4.4 Gut barrier dysfunction: a structural checkpoint connecting dysbiosis to bone loss

The intestinal barrier is a multilayered defence system that restricts bacterial translocation while permitting nutrient absorption. Its integrity depends primarily on the apical junctional complex between intestinal epithelial cells (IECs), particularly tight junctions (TJs) composed of transmembrane proteins—occludin, claudins, junctional adhesion molecules—anchored to the actin cytoskeleton via scaffolding proteins such as zonula occludens (ZO-1, ZO-2, ZO-3) (Allaire et al., 2018). Beyond the epithelial layer, the gut vascular barrier (GVB) constitutes a second checkpoint controlling the entry of bacteria and antigens into the bloodstream (Ciccia et al., 2017). Disruption of these components—termed “leaky gut”—is now recognized as a core hub connecting dysbiosis, immune activation, and bone loss in OP (Collins et al., 2017; Li et al., 2016; Ma et al., 2020) (Figure 3).

**FIGURE 3:**
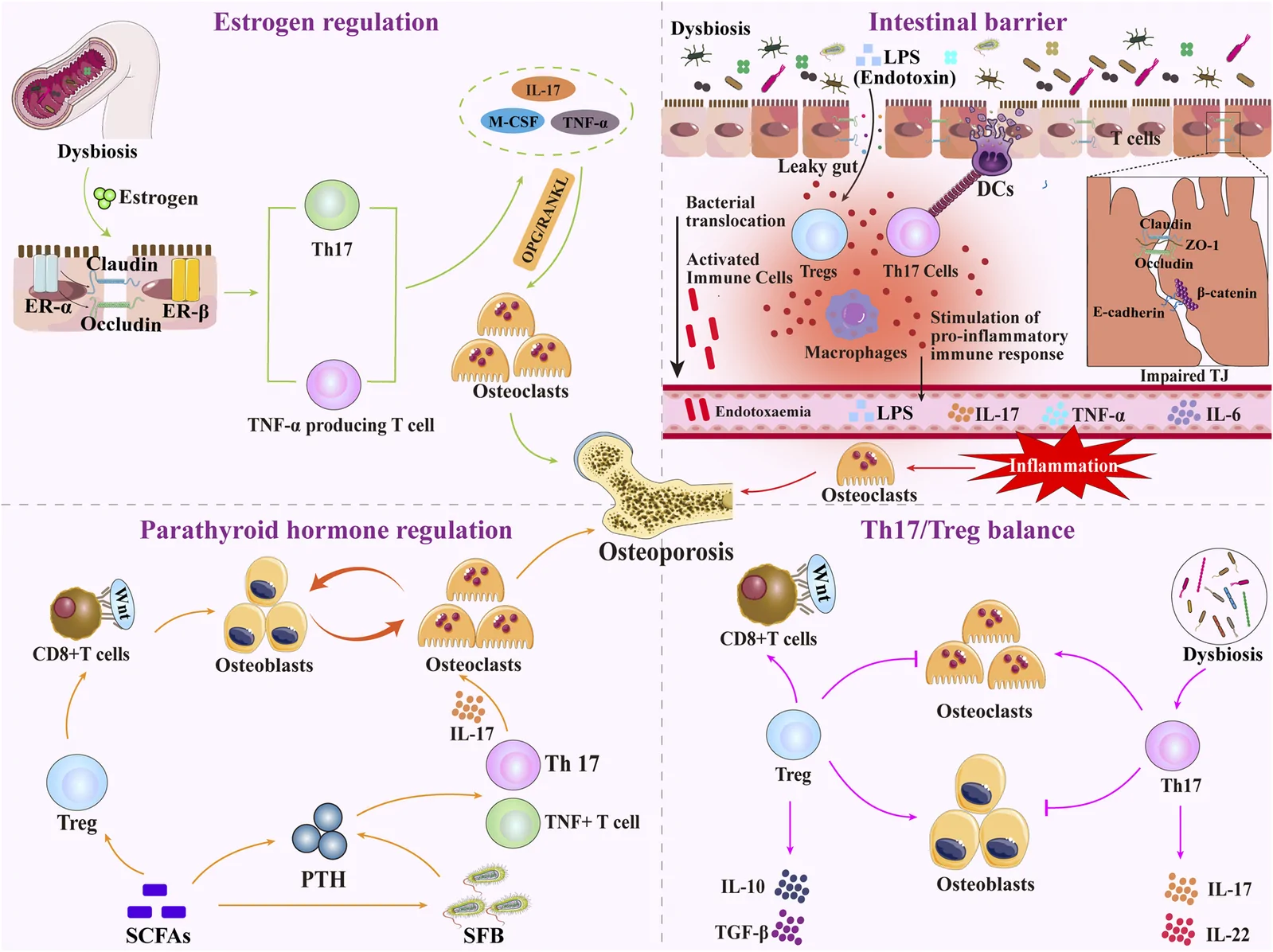
GM regulates the immune-bone axis by mediating hormones and the intestinal barrier. Estrogen deficiency remodels gut microbial profiles, downregulates tight junction proteins occludin, claudin and ZO-1, damages intestinal E-cadherin and epithelial adhesion, and shifts immune cell signaling via ER-α and ER-β toward a pro-inflammatory phenotype; intermittent PTH’s bone-building capacity is entirely dependent on gut microbial SCFAs, whereas SFB trigger expansion of intestinal TNF^+^ T cells and Th17 cells under PTH stimulation, which traffic to bone marrow to boost bone resorption, and SCFAs drive CD8^+^ T cells to produce osteogenic Wnt10b to offset PTH-related bone loss; gut dysbiosis impairs intestinal tight junctions and triggers microbial translocation and systemic LPS-driven endotoxemia, circulating LPS activates TLR4 signaling in dendritic cells and macrophages to release TNF-α, IL-6 and other pro-inflammatory cytokines, elevating the RANKL/M-CSF ratio and inhibiting OPG expression to disrupt normal bone remodeling; gut microbial metabolites govern Th17/Treg immune equilibrium, with SCFAs and secondary BAs promoting Foxp3^+^ Treg differentiation and secretion of protective IL-10 and TGF-β, while SFB and translocated LPS upregulate RORγt to expand IL-17-producing pro-inflammatory Th17 cells, and the resulting imbalanced Th17/Treg ratio amplifies systemic inflammation, accelerates osteoclast formation and suppresses osteoblast function, ultimately facilitating the development of OP.

A growing body of evidence (Tajik et al., 2020) identifies zonulin as a master regulator of paracellular permeability in OP. Zonulin binds to protease-activated receptor 2 (PAR2) on IECs, triggering phosphorylation and disassembly of TJ proteins, notably ZO-1 and occludin. In collagen-induced arthritis mice, serum zonulin rises with disease progression, and pharmacological inhibition of zonulin with Larazotide significantly reduces OC numbers, attenuates joint inflammation, and preserves bone density, demonstrating that zonulin-mediated barrier disruption is a therapeutically tractable driver of bone loss. In PMO (Li et al., 2016), estrogen deficiency may upregulate zonulin expression, thereby facilitating systemic low-grade inflammation—a hallmark of PMO pathogenesis.

Beyond their structural role, certain claudins exert direct, non-canonical effects on bone cells. At the intestinal level, claudin-1, -2, -12, and -15 are consistently downregulated in OVX rats, correlating with increased permeability and enhanced osteoclastogenic cytokine release (Chen W.-J. et al., 2024). Claudin-11 (Cldn11) has emerged as a dual modulator: *Cldn11*-deficient mice exhibit a ∼40% reduction in trabecular bone, and Cldn11 enhances osteogenesis while inhibiting osteoclastogenesis via bidirectional EphB4–EphrinB2 signaling (Baek et al., 2018). Claudin-18 (Cldn-18) acts as a negative regulator of osteoclast differentiation by interacting with ZO-2 to modulate RANKL signaling (Linares et al., 2012). These findings establish that certain claudins are not merely passive structural components but active participants in bone remodeling, providing a direct molecular link between intestinal epithelial integrity and skeletal homeostasis.

Occludin and ZO-1 are consistently downregulated in osteoporotic conditions. In OVX mice and in recipients of fecal microbiota transplantation (FMT) from postmenopausal OP patients, intestinal occludin and ZO-1 expression is significantly reduced, and this reduction parallels increased serum LPS and elevated bone resorption markers (Deng et al., 2026; Zhang Y.-W. et al., 2022). Restoration of occludin and ZO-1 is a common mechanistic endpoint for diverse therapeutic strategies—probiotics (*Lactobacillus rhamnosus* GG, *Bifidobacterium*), prebiotics (lactulose, FOS), FMT, and natural compounds (e.g., berberine, Shengu granules)—all of which improve barrier integrity, suppress systemic inflammation, and prevent bone loss (McKenzie et al., 2017).

LPS, a principal endotoxin of Gram-negative bacteria, transduces barrier disruption into osteoimmune imbalance. When TJ integrity is compromised, LPS traverses the epithelium and activates TLR4 on macrophages, triggering MyD88/NF-κB and TRIF/IRF-3 pathways that promote polarization toward a pro-inflammatory M1-like macrophage phenotype and release of IL-1β, IL-6, and TNF-α—cytokines that directly stimulate osteoclastogenesis and suppress OB function (AlQranei et al., 2021). In postmenopausal women, elevated plasma LPS correlates with increased intestinal permeability, lower BMD, and higher bone resorption markers (Ma et al., 2024). Importantly, LPS synergizes with RANKL to enhance osteoclastogenesis, establishing a vicious cycle in which barrier dysfunction and RANKL-driven resorption reinforce each other.

The GVB represents an emerging, less explored dimension of gut–bone crosstalk. The GVB, composed of specialized endothelial cells expressing PLVAP, controls the passage of microbial products into the portal circulation. In patients with ankylosing spondylitis—a condition frequently associated with OP—GVB impairment is accompanied by zonulin upregulation and elevated serum LPS-binding protein and intestinal fatty acid-binding protein (Ciccia et al., 2017). Although direct evidence in OP remains limited, preclinical studies indicate that intestinal inflammation triggers both GVB dysfunction and bone loss (Liu et al., 2026), suggesting that GVB integrity may be an additional checkpoint modulating the amplitude of inflammatory signals reaching the bone marrow.

In summary, gut barrier dysfunction in OP is not monolithic but involves distinct but interconnected molecular players—zonulin as an upstream disassembler, claudins/occludin/ZO-1 as structural effectors, LPS/TLR4 as the principal inflammatory transducer, and the GVB as a vascular checkpoint. Critically, these components are not merely passive biomarkers; they are active participants in OP pathogenesis. Therapeutic interventions that restore barrier integrity—probiotics, prebiotics, FMT and specific dietary regimens—consistently converge on upregulation of TJ proteins (ZO-1, occludin, claudins), reduction of systemic LPS, and correction of Th17/Treg imbalances and macrophage polarization states (Peng et al., 2009; Singh et al., 2014), thereby breaking the “dysbiosis → leaky gut → endotoxaemia → inflammation → bone loss” cascade. Targeting these barrier components offers a rational, multi-mechanistic approach that addresses upstream drivers of OP rather than merely mitigating terminal bone loss.

## 5 Bone marrow immune microenvironment: a hub for gut-derived immune cell trafficking

The bone marrow (BM) is not merely the primary site of hematopoiesis but also a sophisticated immunological niche where immune cells interact with skeletal cells to orchestrate bone remodeling. Within this microenvironment, hematopoietic stem cells, mesenchymal stem cells, OBs, OCs, and a diverse array of immune cells—including T cells, B cells, macrophages, NK cells, and dendritic cells—form a densely interconnected network that continuously receives input from the intestinal immune compartment via the systemic circulation (Jha et al., 2025). This gut-to-BM immune cell trafficking establishes the BM as the terminal effector site of the gut–immune–bone axis, where signals initiated by GM and metabolites are transduced into osteoclastogenic or osteoprotective outcomes (Ma et al., 2025; Pu et al., 2025).

### 5.1 Physiological trafficking of gut-derived immune cells to the BM

Under steady-state conditions, a continuous but low-grade trafficking of intestinal lymphocytes to the BM occurs, contributing to the maintenance of immune surveillance and hematopoietic niche homeostasis. Intestinal T cells and NK cells egress from gut-associated lymphoid tissues (GALT), particularly Peyer’s patches (PPs) and mesenteric lymph nodes, through S1PR-mediated mechanisms, enter the systemic circulation, and subsequently home to the BM via chemokine receptor–ligand interactions. The S1P/S1PR axis serves as the master regulator of lymphocyte egress: sphingosine-1-phosphate receptor 1 (S1PR1) governs T cell exit, whereas sphingosine-1-phosphate receptor 5 (S1PR5) predominantly regulates NK cell egress (Baeyens et al., 2015; Walzer et al., 2007). Upon entering the circulation, these cells are guided to the BM by constitutively expressed chemokines—including CXCL12, CXCL9, CXCL10, and CCL20—produced by BM stromal cells, OBs, and endothelial cells (Shin et al., 2010). This constitutive trafficking ensures that the BM immune repertoire remains dynamically connected to the gut immune landscape, allowing systemic immune states to influence local bone metabolism.

### 5.2 Pathological amplification of gut-to-BM trafficking in OP

A landmark study by Yu et al. (2021) provided the first direct evidence that pathological bone loss in estrogen deficiency is driven by GM-dependent trafficking of intestinal T cells to the BM. Using Kaede photoconvertible mice to track cell migration *in vivo*, the authors demonstrated that OVX expanded intestinal Th17 cells and TNF^+^ T cells, increased their S1PR1-mediated egress from the intestine, and enhanced their subsequent influx into the BM through a dual chemokine axis: CXCR3 for TNF^+^ T cells and CCL20/CCR6 for Th17 cells. Mechanistically, OVX-induced GM dysbiosis increased intestinal permeability, enabling translocation of bacterial products that activated T cells in the lamina propria. The expanded intestinal TNF^+^ T cells then homed to the BM via CXCR3, where they produced TNF that upregulated BM CCL20 expression, creating a chemotactic gradient that recruited Th17 cells through CCR6. The functional relevance of this trafficking cascade was unequivocally demonstrated by the finding that blockade of T cell egress from the gut (using the S1PR1 functional antagonist FTY720) or blockade of their influx into the BM (via anti-CCL20 antibody or CXCR3 genetic deletion) completely prevented OVX-induced trabecular bone loss.

Notably, this mechanistic paradigm extends beyond estrogen deficiency. Yu et al. (2021) further demonstrated that PTH also induces bone loss through microbial-dependent expansion of intestinal TNF^+^ T cells and Th17 cells, engaging an identical trafficking circuit—S1PR1-mediated egress, CXCR3-guided TNF^+^ T cell influx, and CCL20/CCR6-driven Th17 cell recruitment. Collectively, these observations establish that intestinal T cells are a proximal target of hormonal and metabolic signals relevant to bone homeostasis, and the BM immune microenvironment is the distal effector site where gut-origin T cells execute their osteoclastogenic program.

### 5.3 Gut-to-BM trafficking in bone metastasis: a protective paradigm

Complementing the osteoporotic context, Pal et al. (2022) revealed that gut-to-BM immune cell trafficking can also exert protective functions against bone metastasis. In a B16-F10 melanoma bone metastasis model, the authors showed that GM depletion by broad-spectrum antibiotics accelerated intraosseous tumor growth and osteolysis. Mechanistically, melanoma growth in bone triggered expansion of intestinal NK cells and Th1 cells and promoted their S1P-mediated egress from the gut, followed by CXCR3/CXCL9-mediated influx into tumor-bearing bones. Antibiotic-induced microbiome ablation blunted this trafficking response, preventing the accumulation of NK and Th1 cells in the BM and thereby unleashing tumor progression. Critically, pharmacological blockade of NK cell egress (using the S1PR5 antagonist BAF312), Th1 cell egress (FTY720), or their CXCR3-mediated influx (anti-CXCL9 antibody or Cxcr3^−/−^ mice) each recapitulated the effects of microbiome depletion, accelerating bone tumor growth (Pal et al., 2022). This study established a conceptually novel paradigm: the GM restrains melanoma bone metastasis by promoting the trafficking of antitumor immune cells from the intestine to the BM.

### 5.4 Chemokine axes orchestrating gut-to-BM immune cell trafficking

The trafficking of gut-derived immune cells to the BM is governed by a coordinated network of chemokine receptors and their ligands, which operate at two distinct checkpoints: egress from the GM and influx into the BM.

At the egress checkpoint, S1P/S1PR signaling is the dominant mechanism. S1PR1, expressed on T cells, promotes lymphocyte exit from PPs and mesenteric lymph nodes in response to circulating S1P gradients (Baeyens et al., 2015). S1PR5 serves an analogous function for NK cells. FTY720, a functional S1PR1 antagonist, arrests T cell egress without affecting their function; BAF312 similarly blocks NK cell exit via S1PR5 (Pal et al., 2022; Yu et al., 2021). The differential receptor usage enables selective targeting of specific immune lineages.

At the influx checkpoint, three principal chemokine axes have been identified:

The CXCR3/CXCL9/CXCL10 axis guides the homing of TNF^+^ T cells, Th1 cells, and NK cells to the BM. CXCR3 is expressed on these effector populations, while its ligands CXCL9 and CXCL10 are produced by BM stromal cells and are upregulated under inflammatory conditions (Wendel et al., 2008). In the OVX model, CXCR3 was required for TNF^+^ T cell accumulation in the BM; in the melanoma model, both CXCR3 and its ligand CXCL9 were essential for NK and Th1 cell recruitment. Mechanistically, TNFα-exposed BM mesenchymal stem cells transcriptionally activate CXCR3 ligand chemokines, establishing a chemotactic gradient that attracts circulating effector cells into the marrow space.

The CCL20/CCR6 axis is specifically responsible for Th17 cell homing to the BM. CCR6 is constitutively expressed on Th17 cells (Schutyser et al., 2003), while CCL20 is produced by BM cells in response to TNF stimulation. This creates a feed-forward loop: gut-derived TNF^+^ T cells that arrive in the BM via CXCR3 produce TNF, which upregulates CCL20 expression, thereby recruiting Th17 cells through CCR6. This hierarchical trafficking cascade ensures that Th17 cell accumulation in the BM is contingent upon prior TNF^+^ T cell infiltration.

The CXCL12/CXCR4 axis plays a more general role in BM homing, mediating the retention of various immune progenitors and mature lymphocytes within the BM niche. While not specific to gut-derived cells, CXCL12 provides the constitutive chemotactic background upon which the inducible axes operate.

Collectively, these coordinated checkpoints—S1P–S1PR for egress, CXCR3 and CCL20/CCR6 for influx, and CXCL12/CXCR4 for niche retention—constitute a multilayered trafficking code that determines the composition of the BM immune landscape in response to gut-derived signals.

### 5.5 Integration into the gut–immune–bone axis framework and therapeutic implications

The recognition of gut-to-BM immune cell trafficking as a core mechanism of the gut–immune–bone axis reframes our understanding of how GM regulate skeletal homeostasis. Rather than acting through systemic diffusion of microbial metabolites alone, the gut–bone connection operates through a three-step relay: (1) gut dysbiosis or microbial signals activate and expand specific immune cell populations in the intestinal lamina propria; (2) these cells egress from the gut via S1P/S1PR signaling; and (3) they home to the BM via chemokine receptor–ligand interactions, where they execute their osteoclastogenic or osteoprotective programs within the BM immune microenvironment (Pal et al., 2022; Yu et al., 2020; Yu et al., 2021).

This trafficking-centric model carries three key therapeutic implications. First, it identifies the gut as a proximal and pharmacologically accessible target: interventions that modulate GM composition or intestinal T cell activation can intercept the pathogenic cascade before immune cells reach the BM. Second, it nominates the trafficking machinery itself—S1PR1/S1PR5, CXCR3, and CCR6—as druggable checkpoints. FTY720 (FDA-approved for multiple sclerosis) and BAF312 (siponimod, approved for secondary progressive MS) could potentially be repurposed for skeletal indications (Pal et al., 2022; Yu et al., 2021). Third, it provides a mechanistic rationale for probiotic interventions: by restoring gut barrier integrity and normalizing intestinal immune activation, these strategies may reduce the pool of gut-derived osteoclastogenic T cells available for trafficking to the BM. Notably, recent evidence has extended this paradigm by revealing a bidirectional trafficking circuit: CD8^+^ T cells can traffic between the intestine and BM in a reciprocal manner, with immunogenic chemotherapy redirecting these cells back to the BM through integrin α4–VCAM-1 interactions (Shi et al., 2024).

Importantly, the same trafficking apparatus can produce diametrically opposite outcomes depending on the immunological context: in estrogen deficiency and PTH excess, gut-derived Th17 and TNF^+^ T cells drive bone loss (Yu et al., 2020; Yu et al., 2021); in melanoma bone metastasis, gut-derived NK and Th1 cells restrain tumor growth. This contextual duality underscores the need for lineage-specific targeting rather than global manipulation of trafficking.

In summary, the BM immune microenvironment serves as the terminal integrator of gut-derived immune signals. Elucidating the molecular mechanisms that govern the egress, circulation, and homing of gut-derived immune cells to the BM—and understanding how these processes are differentially regulated across skeletal pathologies—will be essential for developing precision therapies that harness or intercept this trafficking network for skeletal health.

## 6 Subtype-specific heterogeneity of the GM-immune-bone axis: from postmenopausal to secondary osteoporosis

Notably, the regulatory effects of the GM-immune-bone axis are not uniform across all OP patients; rather, they exhibit distinct patterns according to the underlying etiology. Primary osteoporosis is conventionally divided into postmenopausal (Type I) and senile (Type II) subtypes, whereas secondary osteoporosis arises from identifiable conditions such as glucocorticoid therapy or diabetes mellitus. Each subtype displays characteristic GM compositions, immune dysregulation profiles, and gut-barrier phenotypes, with direct implications for therapeutic targeting.

### 6.1 PMO: estrogen-driven Th17/Treg imbalance with high GM dependency

PMO is driven by estrogen withdrawal, which profoundly reshapes both the gut microbial ecosystem and the systemic immune landscape. In PMO patients (Ma et al., 2024), 16S rRNA gene sequencing has revealed decreased bacterial richness and diversity, with significant compositional shifts at both phylum and genus levels—including increased *Bacteroidota, Proteobacteria*, and *Campylobacterota*, alongside reduced *Firmicutes, Butyricicoccus,* and *Faecalibacterium*. Cross-sectional profiling further confirms that PMO cohorts harbor a *Firmicutes-enriched/Bacteroidetes*-depleted signature and lower abundance of butyrate- and propionate-producing taxa such as *Butyricicoccus, Fusicatenibacter*, and *Prevotella histicola*. Conversely, pathobionts, including *Klebsiella, Escherichia*, and certain *Clostridium* species, are expanded. These compositional shifts correlate with circulating bone turnover markers and hip BMD, suggesting that gut microbial signatures may serve as biomarkers or therapeutic targets.

Estrogen deficiency compromises intestinal tight junctions, decreasing the expression of proteins that bind epithelial cells together and enabling LPS translocation. In clinical cohorts (Ma et al., 2024), PMO patients exhibit significantly elevated plasma LPS levels, indicating disrupted intestinal integrity and permeability. This “leaky gut” facilitates systemic inflammation that skews the Th17/Treg balance toward osteoclastogenic Th17 cells while suppressing Tregs. PMO patients show decreased frequencies of Treg cells and reduced circulating IL-10, alongside elevated proinflammatory cytokines and Th17 cells. Moreover, estrogen deficiency compromises the anti-osteoclastogenic and immunomodulatory functions of Bregs, leading to a further decrease in Treg frequencies while increasing Th17 cell frequencies and their associated cytokines.

Critically, this dysbiosis is functionally causal: GF or T-cell-depleted mice do not lose bone after OVX, underscoring the GM-immune axis as a mechanistic core. FMT from healthy donors into ovariectomized mice restores intestinal barrier integrity, rebalances Th17/Treg ratios in both gut and bone marrow, and prevents trabecular bone loss. Probiotic supplementation with *Lactobacillus rhamnosus GG* (Guo et al., 2023), *L. reuteri* (Britton et al., 2014), or *Bifidobacterium longum* (Sapra et al., 2022) recapitulates these benefits: tight-junction proteins ZO-1 and occludin rise, LPS leakage falls, and Treg-derived IL-10 and TGF-β suppress osteoclastogenesis while enhancing osteoprotegerin expression. Collectively, these findings establish that PMO is highly amenable to GM-targeted therapies.

### 6.2 Senile osteoporosis: inflammaging-dominant bone loss with partial GM independence

Senile osteoporosis (SOP) arises from cumulative aging effects rather than a specific hormonal trigger. The GM changes in SOP are more heterogeneous and less pronounced than in PMO. Critically, recent GF mouse studies have fundamentally challenged the universality of the gut-bone axis in age-related bone loss. You et al. (2024) demonstrated that the bone phenotype of 24-month-old GF mice was indistinguishable from that of their littermates colonized by fecal transplant at 1 month of age. After 23 months of treatment, there were no significant differences in trabecular bone volume fraction, cortical thickness, or cortical area between GF and colonized mice in either sex. Moreover, bone loss from 3 to 24 months of age was comparable between GF and specific pathogen-free mice. The effect of microbial colonization on bone phenotypes was independent of the microbiome donors’ age. These findings demonstrate that age-related bone loss occurs independent of the GM.

The dominant immune feature of SOP is inflammaging—chronic low-grade systemic inflammation driven by cellular senescence and the senescence-associated secretory phenotype (SASP)—rather than a defined Th17/Treg imbalance (Cheng et al., 2025). Senescent cells accumulate in multiple tissues, producing SASP factors that impinge on the tissue microenvironment with detrimental local and systemic consequences. The interplay between cellular senescence and GM alterations emerges as a pivotal axis in the aging process, increasingly recognized for its contribution to systemic inflammation, physiological decline, and predisposition to age-associated diseases, including OP. However, unlike PMO, where immune dysregulation is acutely triggered by estrogen withdrawal, SOP involves a gradual, cumulative deterioration of immune homeostasis that is less clearly linked to a single microbial or immunological axis.

These findings suggest that GM-targeted interventions may be less effective in pure SOP compared to PMO, and that anti-inflammaging or senolytic strategies may be more relevant for this subtype.

### 6.3 Secondary osteoporosis: disease-specific GM-immune perturbations

Secondary osteoporosis encompasses conditions such as glucocorticoid-induced osteoporosis (GIOP) and diabetic osteoporosis, each with distinctive GM–immune signatures.

#### 6.3.1 GIOP

Glucocorticoids directly induce OB and osteocyte apoptosis but also alter GM composition. In a murine model, long-term broad-spectrum antibiotic treatment prevented glucocorticoid-induced trabecular bone loss, demonstrating the requirement of GM for GIOP. Treatment with the probiotic *Lactobacillus reuteri* also prevented trabecular bone loss. Microbiota analyses (Schepper et al., 2020) revealed that glucocorticoid treatment changed the abundance of *Verrucomicrobiales* and *Bacteroidales phyla*, with significant differences in *Porphyromonadaceae* and *Clostridiales* operational taxonomic units. Crucially, transplantation of fecal material from glucocorticoid-treated mice into recipient naïve, untreated wild-type mice caused bone loss, establishing dysbiosis as a transmissible mediator of GIOP.

Glucocorticoids also cause intestinal barrier disruption, as evidenced by a 2.4-fold increase in serum endotoxin levels. Enhancement of barrier function with a mucus supplement prevented both glucocorticoid-induced barrier leakage and trabecular bone loss. In bone, antibiotic treatment, probiotic supplementation, or mucus supplementation all reduced glucocorticoid-induced OB and osteocyte apoptosis. Glucocorticoid-induced suppression of Wnt10b in bone was restored by these interventions. These data highlight the previously unappreciated involvement of the GM and intestinal barrier function in trabecular GIOP pathogenesis and identify the gut as a novel therapeutic target for preventing GIOP.

#### 6.3.2 Diabetic osteoporosis

In hyperglycemia-induced OP, GM dysbiosis plays a functionally causal role. Hyperglycemic mice develop low-turnover OP accompanied by reduced GM diversity. FMT from hyperglycemic mice induced OP in recipient mice independent of blood glucose levels. A depletion of *Bifidobacterium pseudolongum* was associated with bone loss, whereas supplementation with either GM of normoglycemic mice or *Bifidobacterium pseudolongum* alleviated OP in hyperglycemic mice (Jiang et al., 2026).

Mechanistically, both hyperglycemic mice and FMT recipients exhibited elevated serum IL-17A levels, and anti-IL-17A antibody treatment mitigated OP in the FMT model. Decanoic acid levels were elevated in the feces of hyperglycemic mice and the serum of FMT recipients; decanoic acid promoted the differentiation of naïve CD4^+^ T cells into Th17 cells, leading to increased IL-17A production. These findings reveal a microbiome dysbiosis-driven decanoic acid/IL-17A axis in hyperglycemia-induced OP. IL-17 functions as an osteoclastogenic cytokine that induces RANKL expression on osteoclastogenesis-supporting cells and OBs, while also promoting the production of inflammatory cytokines such as TNF-α, IL-1, and IL-6 that reinforce RANKL production.

Clinically, these subtype-specific differences mandate stratified therapeutic approaches: PMO is highly amenable to probiotics and FMT; SOP may require anti-inflammaging or senolytic interventions; GIOP calls for barrier-protective strategies and probiotic supplementation; and diabetic osteoporosis may benefit from IL-17-targeted or *Bifidobacterium*-based therapies. Recognizing OP as biologically heterogeneous, with divergent microbial and immune drivers, is essential for advancing precision medicine in bone health.

## 7 Biomarkers of the GM–immune–bone axis: from correlation to clinical translation

The GM–immune–bone axis offers a repertoire of biomarkers beyond conventional bone turnover markers, with potential for early risk stratification, treatment monitoring, and mechanistic readouts. Recent multi-cohort and multi-omics studies have substantially expanded the biomarker landscape, revealing both consistent signatures and context-dependent heterogeneity that must be considered for clinical translation.

Microbial compositional indices, notably the *Firmicutes/Bacteroidetes* (F/B) ratio, have been repeatedly associated with OP. In a cohort of Chinese Han youth (Lai et al., 2024), the dominant phyla were *Bacteroidota* (50.6%) and *Firmicutes* (41.6%), with *Faecalibacterium*, *Bacteroides*, and *Ruminococcus* significantly differing between normal and low BMD groups. However, a systematic review (Ji et al., 2024) of 10 high-throughput sequencing studies found pronounced heterogeneity in taxonomic profiles, with no consistent alpha-diversity difference but significant beta-diversity variation, underscoring the need for standardized protocols. A multi-cohort meta-analysis (Guo et al., 2026) of 341 samples from six public cohorts confirmed that neither osteopenia nor OP patients exhibited significant alpha-diversity differences compared to healthy controls, yet Bray-Curtis distance analysis revealed significant beta-diversity differences among groups. Machine learning classification models achieved accuracies of 70.1%–80.1% for OP across four validation cohorts, identifying distinct GM signatures characterized by depletion of SCFA-producing bacteria.

Genetically informed approaches have further refined causal inference. A study (Guan et al., 2025) leveraging GWAS data from European populations identified that family *Bifidobacteriaceae*, genus *Bifidobacterium*, and species *Bifidobacterium adolescentis* exert causal effects leading to decreased heel bone mineral density, with mediation analyses indicating that these effects are driven by circulating stearidonate (18:4n-3) levels via the n-3 pathway (mediating proportion 77.53%–87.24%). Mendelian randomization studies (Gao et al., 2024) have similarly revealed causal relationships between GM and bone mineral density at multiple skeletal sites. Notably, genetic factors interact with the microbiome: a study of 605 individuals found statistically significant interactions between polygenic risk score for OP and taxa such as *Roseburia faecis* and *Megasphaera elsdenii*, with *Roseburia faecis* showing a negative association with BMD specifically in the low-genetic-risk group.

More promising are metabolite biomarkers. Reduced circulating and fecal SCFAs—particularly propionate and butyrate—correlate with low BMD and elevated resorption markers. Direct supplementation in humans decreases serum CTX-I (Lucas et al., 2018). In diabetic osteoporosis models, functional enrichment analysis revealed impaired SCFA metabolism—specifically reduced propanoate and butanoate pathways—associated with decreased bone mass. A Beijing community-based study (Wang Z. et al., 2025) of 76 postmenopausal women demonstrated that *Roseburia_intestinalis* and glycoursodeoxycholic acid were positively associated with BMD. Integrated multi-omics in fracture patients identified 127 differential metabolites, with *Lachnospira eligens* unexpectedly enriched in low bone mass patients despite its prior association with gut health, highlighting context-dependent roles of typically beneficial species (Zhao et al., 2025).

Secondary BAs have emerged as additional metabolite candidates. A study (Zheng X. Q. et al., 2025) demonstrated that GM preserves bone mass through modulation of the hyodeoxycholic acid–TGR5 axis. Integrated metabolomics identified 20 potential PMO biomarkers, with 12 metabolites quantified in amino acid, energy, and BA metabolism pathways. Hyocholic acids were found to play essential roles during OP occurrence and may serve as potential markers. TMAO is negatively correlated with BMD and promotes osteoclastogenesis via NF-κB. In overweight and obese patients, regression analysis found TMAO negatively correlated with whole-body, lumbar spine, and femoral neck BMD, and positively correlated with osteoporotic fracture incidence.

Tryptophan metabolites represent a newly recognized class of axis-derived biomarkers. A study (Bai et al., 2026) identified IPA—a metabolite produced by *Clostridium sporogenes*—as a key regulator of bone-fat balance. IPA levels were significantly reduced in ovariectomized mice and positively correlated with bone mass. Mechanistically, IPA modulates PPARγ signaling to reprogram BMSC lineage commitment from adipogenesis toward osteogenesis. This finding establishes IPA as a previously under-recognized GM-derived metabolite that maintains skeletal homeostasis through suppression of PPARγ signaling.

Immune cell and cytokine signatures form a second pillar. The Th17/Treg ratio, along with IL-17, TNF-α, and the RANKL/OPG ratio, is consistently elevated in osteoporotic patients and mechanistically linked to GM-dependent bone loss (Li et al., 2016). A bidirectional Mendelian randomization analysis (Zhang K. et al., 2025) of 41 inflammatory cytokines found that higher tumor necrosis factor-related apoptosis-inducing ligand (TRAIL) was causally associated with increased OP risk (OR = 1.12). IL-8, IL-17, and IL-22 have been proposed as main biomarkers for PMO due to their ability to stimulate osteoclastogenesis and bone resorption. Elevated IL-17 levels in osteoporotic women and their correlation with fracture risk probabilities suggest a potential role for this cytokine in risk assessment. However, circulating RANKL measurements are confounded by paracrine actions and assay instability, with observational studies yielding inconsistent results regarding the RANKL/OPG ratio and BMD.

Barrier integrity markers—serum zonulin, LPS, and intestinal fatty acid-binding protein (I-FABP)—provide indirect readouts of gut permeability, with elevated LPS correlating with lower BMD. In fracture patients with low bone mass (Zhao et al., 2025), integrated analysis revealed a strong correlation between *Lachnospira eligens* enrichment and inflammation-associated metabolites, including N-acetylneuraminate, suggesting that microbial–metabolite networks may serve as non-invasive diagnostic biomarkers.

The “oral–gut–bone axis” has emerged as an extension of the biomarker paradigm. Metagenomic sequencing of postmenopausal women stratified by BMD revealed significant compositional and functional alterations in the oral microbiome associated with bone mineral density status. A machine learning model integrating oral microbiota (genus *Abiotrophia*) with clinical features achieved an AUC of 0.885 for predicting OP and bone loss in high-altitude Tibetan populations, highlighting the potential of oral factors as non-invasive screening tools (Wang et al., 2026).

Clinical translation faces substantial barriers: cross-sectional data cannot establish causality, inter-individual variability confounds universal thresholds, and standardization across platforms is lacking. Prospective validation against hard fracture endpoints is absent, and cost-effectiveness is uncertain. Nevertheless, machine learning models integrating metagenomic, metabolomic, and immune panels now predict BMD status with 70%–80% accuracy (Guo et al., 2026), and multi-omics unsupervised learning approaches using deep latent space fusion models are being developed to construct comprehensive OP multi-omics atlases. Larger-size longitudinal cohorts based on multi-omics and extended phenotypes are recommended to comprehensively characterize the involvement of GM in OP pathogenesis. These biomarkers are not intended to replace DXA but to complement it, enabling early interception and mechanistic monitoring of GM-targeted therapies.

## 8 Intervention strategies based on the GM-immune-bone axis

Several GM-targeted intervention strategies, including probiotics, prebiotics, fecal microbiota transplantation, and dietary modulation, have been developed to modulate the gut-immune-bone axis (Figure 4).

**FIGURE 4:**
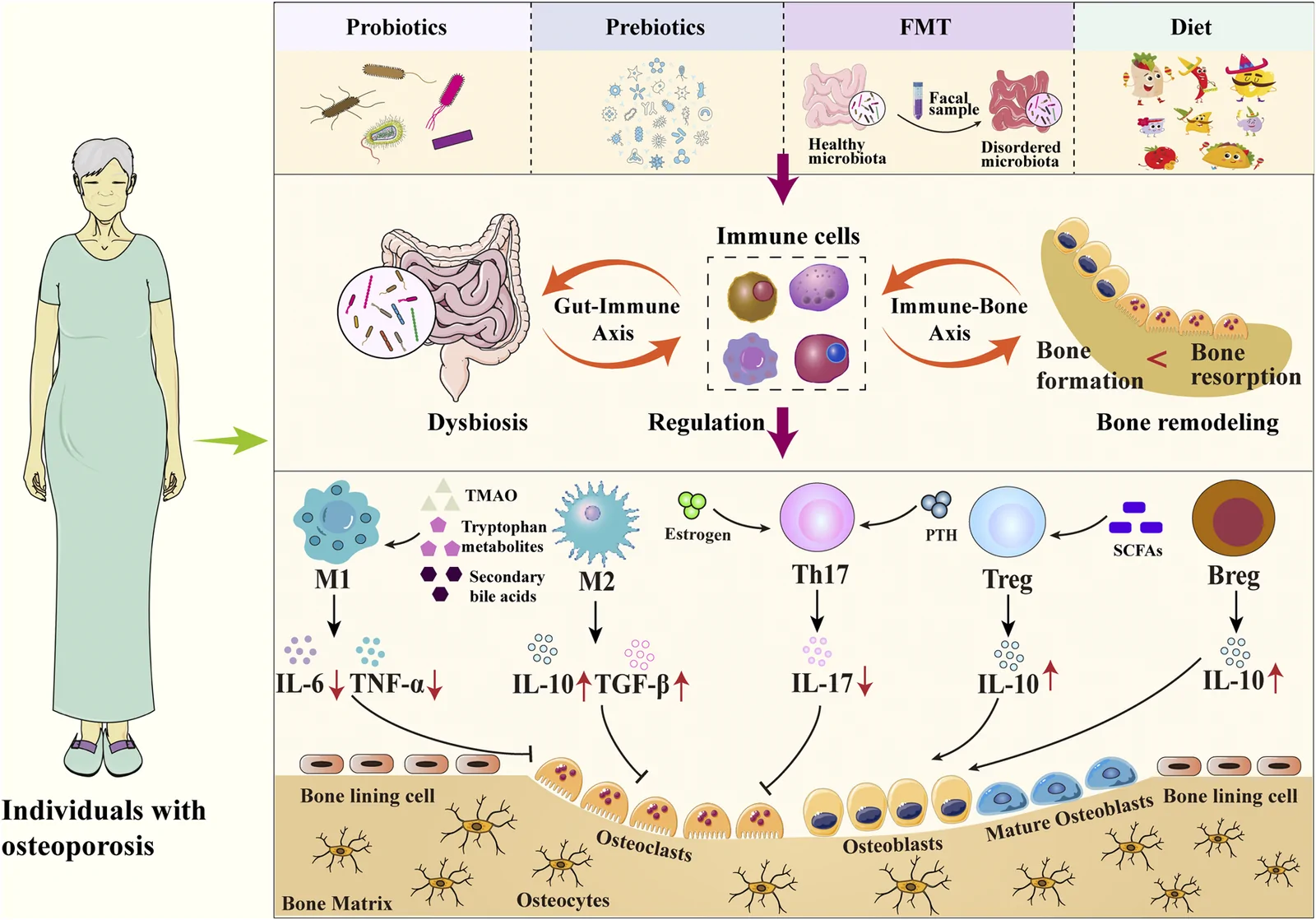
Therapeutic strategies for OP targeting the gut-immune-bone axis. Therapeutic interventions targeting the gut-immune-bone axis include probiotics, prebiotics, FMT, and dietary regulation. These strategies restore GM homeostasis, elevate beneficial microbiota and metabolites such as SCFAs, correct immune imbalance involving Th17/Treg, macrophage polarization states [pro-inflammatory (“M1-like”) versus reparative (“M2-like”)], and Bregs, inhibit bone resorption, and promote bone formation, thereby representing promising approaches for the prevention and treatment of OP.

### 8.1 Probiotics

Probiotics have become a significant milestone in the prevention and treatment of OP. Probiotics primarily regulate OP through mechanisms such as modulating the composition of the GM, regulating the production of inflammatory factors, modulating the immune system, altering metabolites, regulating the endocrine system, and improving intestinal barrier permeability (Bron et al., 2012). Recently, Yu et al. (2022) found that *Lactobacillus* brevis can reduce the levels of pro-resorptive factors such as IL-1, IL-6, and IL-17, suggesting that it may serve as a natural alternative intervention for OP. In a study by Xia et al. (2022), it was observed that *Lactobacillus reuteri ATCC PTA 6475* suppressed TNF-α miRNA levels in the jejunum and ileum of non-pathogen-specific healthy male mice, downregulated IL-1β and IFN-γ expression, and significantly increased bone density.

The Treg/Th17 imbalance is not only the basis of immune homeostasis disruption but also a key factor in the disruption of bone homeostasis. Studies have confirmed (Li et al., 2016) that the probiotic *Lactobacillus rhamnosus GG* promotes Treg secretion of IL-10 and TGF-β by reducing TNF-α, IL-17, and RANKL expression and downregulating Th17 cell numbers, thereby reshaping the immune microenvironment and alleviating bone loss in OVX mice (Guo et al., 2023). Furthermore, *Lactobacillus plantarum GKM3* and *Lactobacillus paracasei GKS6* can both upregulate the expression of osteogenic marker genes such as BMP-2 to enhance osteogenic differentiation, and downregulate the expression of RANK and c-Fos, thereby inhibiting the activity of the OC-associated gene TRAP and suppressing bone resorption (Yang et al., 2020). It is worth noting that the regulation of the Treg/Th17 balance by probiotics exhibits heterogeneity: *Lactobacillus casei CCFM1074* upregulates the Treg/Th17 ratio by promoting the production of SCFAs, particularly butyrate, thereby improving arthritis in CIA rats (Fan et al., 2021); whereas *Lactobacillus casei CCFM1075* primarily reduces the number of Th17 cells.

Probiotics can also exert their effects by secreting metabolites. For example, under estrogen-deficient conditions, *Bacillus coagulans* enhances the anti-osteoclastogenic and immunomodulatory potential of Bregs by inducing GM to produce SCFAs, suggesting that it can alleviate postmenopausal bone loss by regulating the gut-immune-bone axis (Sapra et al., 2025b). Other studies have shown that the indole derivative melatonin significantly increases SCFA production and reduces levels of TMAO-related metabolites. By restoring the balance between pro-inflammatory (“M1-like”) and reparative (“M2-like”) macrophage phenotypes and lowering pro-inflammatory factor levels, it alleviates OP-related clinical symptoms and reverses GM dysbiosis, further confirming the intervention potential of the GM-metabolite-immune network (Chen Y. et al., 2024). Other studies (Schepper et al., 2019) suggest that *Lactobacillus reuteri* prevents bone loss by reversing antibiotic-induced dysbiosis and restoring intestinal barrier integrity, highlighting the importance of GM-regulated intestinal permeability for bone health.

In a clinical study (Takimoto et al., 2018), oral administration of *Bacillus subtilis C-3102* significantly increased hip bone mineral density in postmenopausal women and reduced levels of the bone resorption markers urinary NTX and TRACP, while the abundance of beneficial bacteria such as *Bifidobacterium* increased significantly in the treatment group. Furthermore, Lei et al. (2016) demonstrated in a prospective, double-blind, placebo-controlled randomized clinical trial that for elderly patients with distal radius fractures, supplementation with *Lactobacillus casei* and *Lactobacillus paracasei* probiotics can accelerate the bone healing process, suggesting that probiotics have a positive effect on bone formation. Jones et al., (2013) found that *Lactobacillus reuteri* capsules significantly increased patients’ serum 25-hydroxyvitamin D3 levels, promoting calcium absorption and increasing bone density. These studies collectively demonstrate that probiotics have a positive effect on OP in the elderly.

In summary, probiotics can target the “gut-immune-bone” axis from multiple dimensions—including regulating GM composition, correcting immune imbalances, suppressing inflammatory responses, improving the intestinal barrier, and promoting nutrient absorption—to maintain bone health. However, current research is largely confined to animal studies, with limited large-scale clinical trials. Key issues—such as the optimal probiotic strains, dosage, treatment duration, timing of intervention, and preventive use in high-risk populations—remain unresolved, necessitating further validation through high-quality, large-scale studies.

### 8.2 Prebiotics

Prebiotics are dietary supplements that promote the proliferation of beneficial bacteria such as *Bifidobacterium* and *Lactobacillus* by providing metabolic substrates. This increases the production of beneficial components such as SCFAs, regulates the immune system and calcium absorption and metabolism, and maintains bone metabolic balance. Common prebiotics include lactulose, fructooligosaccharides, galactooligosaccharides, inulin, glucan, and xylan. Chen et al. (2019) found that lactulose altered the GM composition in OVX mice, increased SCFA levels, reduced TNF-α, IL-6, RANKL, and IL-17, and increased IL-10 and Treg cell counts. Studies have shown that after feeding mice with fructooligosaccharides, significant growth and proliferation of *Bifidobacterium* can be observed in the mouse colon, and the higher the dose of fructooligosaccharides, the more significant the increase in the relative abundance of *Bifidobacterium* in the gut. Tousen et al. (2019) observed an increase in the number of *Bifidobacterium* in the intestines of mice fed resistant starch; furthermore, this diet increased the expression of the IL-10, reduced RANKL expression, and alleviated estrogen-deficient bone loss. Similarly, administering 10 g or 20 g of soluble corn fiber daily to healthy adolescent female subjects for 4 weeks increased calcium absorption by approximately 15% (Whisner et al., 2016). A study by Ai et al. (2024) demonstrated that a diet containing 8% konjac oligosaccharides increased trabecular bone count, improved bone flexural stiffness, and mitigated bone loss in OVX rats. The mechanism underlying these effects is associated with the repair of the intestinal barrier, reduced release of pro-inflammatory factors, maintenance of the Treg/Th17 balance in bone marrow, and decreased OC activity.

Prebiotics can also improve bone metabolism by promoting the production of SCFAs. For example, short-chain fructo-oligosaccharides s increase serum butyrate levels and CTX-I (a marker of bone formation) in OVX rats, while having no significant effect on markers of bone resorption (Porwal et al., 2020). Furthermore, it was found that mushroom fermentation products significantly increased the number of *Bifidobacteria* and *Lactobacilli* in postmenopausal women, significantly increased SCFA production, and promoted OB activity (Kerezoudi et al., 2021). Studies (Wang L. et al., 2025) have shown that these SCFAs can enhance Breg activity, maintain immune tolerance, and suppress excessive inflammatory responses, while promoting the activation and proliferation of intestinal lamina propria macrophages, dendritic cells, and Tregs, thereby regulating the balance between anti-inflammatory and pro-inflammatory cytokines.

In summary, probiotics can serve as a future strategy for the prevention and treatment of OP by modulating the GM and its metabolites, correcting Th17/Treg immune imbalance, and improving mineral bioavailability.

### 8.3 FMT

FMT refers to the transfer of functional microbiota from a healthy donor into the recipient’s gastrointestinal tract to improve dysbiosis and restore GM diversity, thereby treating disease. Recent studies (Li et al., 2021) have shown that the efficacy of FMT in treating OP primarily involves improving intestinal barrier function, correcting GM dysbiosis, increasing levels of SCFAs in feces, and modulating immune responses to regulate bone metabolism. Animal studies (Zhang Y.-W. et al., 2022) have demonstrated that FMT upregulates tight junction proteins—such as ZO-1—and inhibits the release of pro-osteocytic cytokines, including TNF-α and IL-1β. Furthermore, FMT has been observed to increase the abundance of beneficial bacteria (including *Prevotella, Lactobacillus*, and *Ruminococcus*) and reduce that of pro-inflammatory bacteria (*E. coli*, *Bacteroides desulfetigenes*), thereby promoting high levels of SCFA production. These SCFAs play a key role in inhibiting osteoclastogenesis and can effectively alleviate OVX-induced bone loss. Specifically, SCFAs (particularly acetate and propionate) inhibit osteoclastogenesis by downregulating RANKL and reducing pro-inflammatory cytokines. Furthermore, FMT can rebalance immune dysregulation in OVX models by upregulating Foxp3 via SCFA-mediated mechanisms and inhibiting Th17 polarization, thereby reducing OC activity. The aforementioned studies indicate that FMT improves OP by precisely regulating the Treg/Th17 balance through improvements in GM composition and metabolism. Overall, FMT represents a promising alternative therapy and a potential future strategy for the prevention and treatment of PMO; larger-scale randomized trials are necessary to verify its long-term efficacy and its ability to reduce fracture risk.

Despite promising preclinical efficacy, the comparative effectiveness of FMT regimens—donor composition, infusion frequency, and treatment duration—remains poorly defined. Evidence from OVX models indicates that multi-session protocols (three infusions over 2–3 weeks) yield superior and more durable bone protection than single or abbreviated courses, and donor stool enriched with butyrate-producing *Clostridium clusters IV/XIVa* exerts stronger osteoprotective effects than age-matched or low-richness preparations. Optimal regimens are subtype-dependent: for PMO, an intensive induction (three infusions within 2 weeks plus monthly maintenance) effectively corrects Th17/Treg skewing; for SOP, lower-frequency FMT (every 3 months) combined with senolytic adjuvants is more appropriate, given the partial GM independence of age-related bone loss; for GIOP, early FMT initiation (within 1 week of steroid exposure, three daily infusions with *Lactobacillus reuteri*-enriched material) prevents barrier disruption and osteocyte apoptosis; and for diabetic osteoporosis, weekly FMT for 4 weeks from normoglycemic donors reduces IL-17A, but requires concurrent glycemic control to sustain efficacy. For high-risk populations, tailored approaches are recommended: (1) severe PMO (T-score < −2.5 with fracture) may benefit from FMT plus low-dose teriparatide; (2) frail elderly (≥75 years) should receive a single FMT followed by maintenance prebiotics to avoid over-immunostimulation; (3) long-term glucocorticoid users warrant prophylactic FMT (one pre-steroid and one at week 2) combined with tight-junction protectors; and (4) antibiotic-associated bone loss responds to a short high-dose course (daily for 5 days) with standardized donor preparations. These observations underscore that personalized FMT regimens—stratified by etiology, donor quality, and intervention timing—are essential for clinical translation, yet rigorous standardization and large-scale trials are urgently needed to confirm long-term fracture-risk reduction.

### 8.4 Dietary regulation

Dietary patterns exert divergent, context-dependent effects on the gut-immune-bone axis, each carrying a distinct risk–benefit profile that must be carefully weighed in clinical recommendations (Zmora et al., 2019). Dietary fiber offers the most robust bone-protective evidence: it enriches SCFA-producing taxa (*Bifidobacterium, Lactobacillus, Faecalibacterium*), promotes Treg differentiation and suppresses Th17 polarization via GPR43/109A (Behler-Janbeck et al., 2024; Singh et al., 2014), reinforces intestinal barrier function (Peng et al., 2009), and directly inhibits osteoclastogenesis while enhancing OB activity (Lou et al., 2024; Zhang H. et al., 2022). However, excessive insoluble fiber (>50 g/day) may impair calcium absorption (Alexander et al., 2022; Wongdee et al., 2021), and the benefit is fiber-type-specific—pectin and inulin outperform cellulose—while rapid fermentation can cause bloating in susceptible individuals (Lou et al., 2024). The Mediterranean diet—rich in polyphenols, omega-3 fatty acids, and fermentable substrates—similarly fosters SCFA production, activates AhR signaling to modulate Treg/Th17 balance (Chen C. et al., 2024; Rahul and Tracy, 2018), and is associated with reduced hip fracture risk in cohort studies (Peng et al., 2024); yet its adherence is challenging outside Mediterranean regions, and phytates in nuts/legumes modestly reduce calcium bioavailability, while the wine component must be strictly limited. Conversely, the ketogenic diet represents a double-edged sword: although ketone bodies may suppress NLRP3 inflammasome activity, the disadvantages are substantial—reduced calcium intake and increased urinary excretion, decreased SCFA production and IGF-1 levels (Li et al., 2020), impaired OB function, and clinical evidence of lower BMD in long-term users—making it inadvisable for OP-prone populations unless accompanied by aggressive supplementation and monitoring. High-protein diets stimulate IGF-1 and preserve muscle mass, but excessive animal protein increases acid load and urinary calcium loss unless offset by abundant alkali-rich vegetables; plant-based proteins with adequate fiber offer a safer balance. Caloric restriction and intermittent fasting reduce oxidative stress but may elevate cortisol and lower leptin, with human data showing decreased BMD and increased resorption, thus not universally recommended, especially in the elderly. Importantly, beyond specific dietary patterns, the overall antioxidant capacity of the diet has been linked to bone health: a recent cross-sectional study (Cui et al., 2024) in children and adolescents aged 8–19 years demonstrated that higher composite dietary antioxidant index (CDAI) was positively correlated with BMD, underscoring the potential of antioxidant-rich foods (fruits, vegetables, whole grains) to support skeletal development from an early age. In summary, the optimal dietary strategy for OP should prioritize a Mediterranean-style, high-fiber regimen (20–35 g/day) with diverse plant proteins and abundant antioxidants, while strictly avoiding ketogenic and unbalanced high-protein patterns (Zhang H. et al., 2022). Personalized adjustments based on baseline microbiota, genetic polymorphisms, and comorbidities are essential, and dietary modulation should be integrated with pharmacotherapy as a foundational, non-invasive component of comprehensive management.

## 9 Synergistic pharmacological mechanisms and clinical prospects of drug–GM combinations in OP

Conventional OP pharmacotherapy—anti-resorptive, anabolic, dual-action, and novel agents—is increasingly recognized as operating within a gut–immune–bone network. Their efficacy and safety are profoundly modulated by the composition and metabolic output of the GM, creating a strong rationale for combination strategies that simultaneously address bone metabolic defects and upstream microbial–immune dysregulation.

### 9.1 Anti-resorptive agents: counteracting GM-driven limitations

Bisphosphonates and denosumab suppress OC activity but induce GM dysbiosis—depleting butyrate-producing taxa and compromising intestinal barrier integrity—which paradoxically sustains low-grade endotoxemia and Th17-skewed inflammation that counteracts their bone-protective effects (Talaat et al., 2015). Adjuvant probiotics (e.g., *Lactobacillus rhamnosus GG*, *Bifidobacterium longum*) or prebiotics restore SCFA production, reinforce tight junctions, reduce systemic LPS, and amplify the anti-inflammatory (IL-10↑, IL-17↓) and anti-osteoclastogenic actions of these drugs. For denosumab, this combination holds particular promise in mitigating the post-withdrawal rebound bone loss, as microbial metabolites maintain Treg numbers and suppress the surge in OPCs upon drug cessation. For SERMs and HRT, GM restoration (*Prevotella histicola, Bifidobacterium*) can partially recapitulate estrogen’s immunomodulatory effects, potentially allowing dose reduction and improved safety profiles in patients with contraindications.

### 9.2 Anabolic agents: exploiting absolute GM dependency

Teriparatide and abaloparatide exhibit an absolute requirement for gut-derived butyrate to drive their bone-forming cascade: butyrate expands bone marrow Tregs, which then stimulate CD8^+^ T-cell Wnt10b secretion and canonical Wnt/β-catenin activation in OBs (Li et al., 2020). Antibiotic eradication or germ-free status completely abrogates iPTH-induced bone formation, establishing butyrate as a non-redundant “permissive signal.” Therefore, co-administration with butyrogenic probiotics (*Clostridium butyricum, Faecalibacterium prausnitzii*) or prebiotic fibers (resistant starch, inulin) acts as a “sensitization” strategy, enhancing anabolic efficacy—potentially enabling shorter treatment courses or lower doses without compromising fracture prevention. This approach is especially relevant for “poor responders” with low baseline fecal butyrate, and may improve the cost-effectiveness and adherence to daily injection regimens.

### 9.3 Dual-action and novel agents: emerging crosstalk and dose-sparing opportunities

Romosozumab (anti-SOST) activates Wnt/β-catenin while suppressing RANKL (Fusaro and Tripepi, 2023). Preliminary evidence indicates that secondary BAs—modulated by the GM—can cross-talk with Wnt signaling via FXR/TGR5, potentially amplifying or antagonizing romosozumab’s effects. Combining it with prebiotic inulin or *Akkermansia muciniphila* may not only augment BMD gains within the narrow 12-month window but also mitigate the pro-inflammatory milieu linked to cardiovascular concerns, though this requires rigorous testing.

Novel anti-RANKL biologics (e.g., narlumosbart) and cathepsin K inhibitors offer refined targeting. GM metabolites, particularly butyrate and indole derivatives, directly suppress RANKL-induced NFATc1 and c-Fos expression, creating synergistic/additive inhibition of osteoclastogenesis when combined with subtherapeutic antibody doses. This opens the door to dose-sparing strategies—reducing drug exposure, cost, and potential immunogenicity—while maintaining efficacy. Conceptually, anti-IL-17 antibodies combined with Treg-promoting prebiotics could deliver dual checkpoint correction of the Th17/Treg imbalance, offering a disease-modifying potential beyond BMD improvement, although this remains speculative.

### 9.4 Translational roadmap and future paradigm

Clinical translation of these synergies requires a biomarker-stratified approach targeting four priority populations: (1) women with elevated LPS/hs-CRP for probiotic-enhanced anti-resorptives; (2) teriparatide users with low butyrate for prebiotic “rescue”; (3) denosumab recipients at rebound risk for GM support during transition; and (4) frail elderly for FMT to restore anabolism. Factorial trials with co-primary endpoints (BMD, fractures, SCFAs, Th17/Treg, Wnt10b) and baseline metagenomic profiling are essential for validation and responder pre-stratification. Safety dictates pharmaceutical-grade strains and strict pathogen screening, especially in immunocompromised hosts. Ultimately, humanized gnotobiotic models and AI-driven multi-omics will enable personalized drug–microbe pairing, transforming GM modulation from adjunct to integral therapy. This “pharmaco-microbiome” synergy paradigm promises enhanced efficacy, reduced toxicity, and durable fracture prevention—now awaiting rigorous, mechanism-guided RCTs for clinical adoption.

## 10 Discussion and perspectives

The GM-immune-bone axis established in this review represents a paradigm shift from bone-centric or dual-axis models toward an integrated tripartite regulatory network. Its core innovation lies in the identification of immune cells as the indispensable central bridge, with exclusive triple-junction nodes—including the GM dysbiosis–Th17/Treg checkpoint and the intestinal barrier–LPS inflammatory relay—that cannot be explained by prior two-organ frameworks (Li et al., 2016; Yu et al., 2021). Mechanistically, this axis operates through four signature pathways: SCFA–GPR43/AhR–RORγt/Foxp3–RANKL/OPG (Behler-Janbeck et al., 2024; Li et al., 2020), secondary BA–TGR5–M2–Wnt10b (Chen C. et al., 2024), LPS–TLR4–NF-κB–NFATc1 (Li et al., 2016), and tryptophan metabolite–AhR–Breg pathways (Sapra et al., 2025b), all of which depend on the co-participation of GMs, immune cells, and bone cells.

Despite these conceptual advances, critical unresolved questions persist. Causality between specific microbial taxa and OP phenotypes remains largely correlational, as most differentially abundant species identified in cross-sectional studies lack functional validation through mono-colonization or targeted depletion, and strain-level heterogeneity within genera such as *Lactobacillus* and *Bifidobacterium* remains unexplored (Guan et al., 2025; Wang Z. et al., 2025). The spatiotemporal dynamics of GM metabolites in bone are poorly defined—the precise concentrations of SCFAs, secondary BAs, and tryptophan derivatives reaching the bone marrow niche, the relative contribution of local versus systemic pools, and the receptor pleiotropy (GPR41/43/109A, AhR, FXR, TGR5) with concentration-dependent duality remain unresolved (Behler-Janbeck et al., 2024; Chen C. et al., 2024; Li et al., 2020). Furthermore, the mechanistic hierarchy among dysbiosis, immune imbalance, and bone loss has not been established; whether dysbiosis is the primary trigger or a secondary consequence in different OP subtypes remains a “chicken-and-egg” conundrum that demands longitudinal interventional studies (You et al., 2024).

Critically, subtype-specific heterogeneity dictates that the axis does not operate uniformly across all OP patients (Table 5). In PMO, estrogen withdrawal drives a highly GM-dependent cascade characterized by Th17 expansion, Treg reduction, and compromised gut barrier—a phenotype highly amenable to probiotics and FMT (Guo et al., 2023; Li et al., 2016). In contrast, SOP exhibits bone loss that occurs largely independently of the GM in GF mice, with inflammaging—driven by cellular senescence and the SASP—emerging as the dominant immune feature (Cheng et al., 2025). GIOP shows an absolute requirement for GM and barrier integrity, as antibiotic treatment or mucus supplementation prevents steroid-induced bone loss (Schepper et al., 2020), whereas diabetic OP is driven by a GM–decanoic acid–IL-17A axis transmissible via FMT independently of glycemic status (Jiang et al., 2026). These divergent trajectories mandate stratified therapeutic approaches: PMO for probiotics/FMT; SOP for senolytic or anti-inflammaging strategies; GIOP for barrier-protective interventions; and diabetic OP for IL-17-targeted or *Bifidobacterium*-based therapies.

**TABLE 5:** Summarizes the comparative GM and immune characteristics across the three major OP subtypes.

Feature	PMO (type I)	SOP (type II)	GIOP	Diabetic OP
Primary driver	Estrogen deficiency	Cellular senescence	Glucocorticoid therapy	Hyperglycemia/insulin resistance
Microbial diversity	Markedly decreased	Moderately decreased	Altered (specific taxa shifts)	Decreased
Key microbial shifts	↓ Firmicutes/↑ Bacteroidetes; ↓ butyrate producers (Butyricicoccus, Prevotella); ↑ *Klebsiella*, *Escherichia*	Heterogeneous; less consistent than PMO	↑ Verrucomicrobiales, Bacteroidales; altered Clostridiales	Reduced diversity; FMT transmits phenotype
Microbiota dependency	High (GF mice protected from OVX bone loss)	Low (bone loss independent of microbiome in GF mice)	High (ABX prevents GIO; FMT transmits GIO)	High (FMT transmits OP independent of glucose)
Dominant immune feature	Th17 expansion, Treg reduction	Inflammaging (SASP)	Barrier injury + endotoxemia	IL-17A axis activation
Th17/Treg balance	Skewed toward Th17	Less pronounced skewing	Complex (GCs are immunosuppressive)	IL-17-mediated
Gut barrier status	Compromised (reversible with estrogen)	Declines with age/comorbidities	Severely disrupted (causal mediator)	Impaired
Therapeutic implications	Probiotics, FMT, estrogen	Anti-aging, anti-inflammatory	Barrier protection, probiotics	Metabolic control, GM-targeted

The translational gap between murine models and human pathophysiology remains a major barrier. Fundamental differences in microbial ecology, immune ontogeny, and environmental exposures limit direct extrapolation of preclinical findings, and the absolute GM dependency of PTH-induced bone anabolism demonstrated in GF mice has not been rigorously tested in humans. The long-term safety of FMT—particularly the risk of transmitting multidrug-resistant organisms—remains inadequately addressed, and standardized donor screening protocols are urgently needed (Li et al., 2021). Optimal regimens (donor composition, multi-session vs. single infusion, treatment duration) remain poorly defined, though emerging evidence from OVX models suggests that multi-session protocols and donor stool enriched with butyrate-producing *Clostridium clusters IV/XIVa* confer superior osteoprotective effects (Zhang Y.-W. et al., 2022).

The axis offers promising biomarker opportunities for early screening and treatment monitoring. Microbial compositional indices—particularly the *Firmicutes/Bacteroidetes* ratio and depletion of SCFA-producing taxa—correlate with low BMD and may predict fracture risk independently of DXA (Guo et al., 2026; Ji et al., 2024; Lai et al., 2024). Metabolite panels, including circulating and fecal SCFAs (especially propionate and butyrate), secondary BAs, and TMAO, reflect both osteoclastogenic activity and barrier integrity (Bai et al., 2026; Lucas et al., 2018; Zheng X. Q. et al., 2025). Immune indices—such as the Th17/Treg ratio and macrophage polarization status [e.g., the relative abundance of pro-inflammatory (“M1-like”) versus reparative (“M2-like”) phenotypes]—provide a dynamic readout of the osteoimmune set-point (Sapra et al., 2025b). Notably, shifts in these parameters often precede measurable BMD changes, positioning them as early surrogates of therapeutic efficacy. However, clinical translation faces substantial obstacles: causality remains unproven, inter-individual variability confounds universal cut-offs, standardization across platforms is lacking, and prospective validation against hard fracture endpoints is absent. Machine learning models integrating metagenomic, metabolomic, and immune panels now predict BMD status with 70%–80% accuracy, offering a roadmap toward composite, actionable biomarker panels (Guo et al., 2026).

Looking further ahead, several frontier directions warrant particular attention to deepen mechanistic understanding and accelerate clinical translation. First, the neuroendocrine interface of the gut-immune-bone axis remains largely uncharted. Gut-derived serotonin, dopamine, and neuropeptides—modulated by specific microbial taxa—can signal through the enteric nervous system and vagal afferents to influence hypothalamic-pituitary axes, thereby indirectly regulating bone turnover. Deciphering this gut–brain–bone circuit will complement the immune-centric model and unveil new neural targets for OP intervention, particularly in stress- and depression-associated bone loss where the GM–immune–neural interplay may be paramount (Ibrahim et al., 2022). Second, spatial and single-cell multi-omics technologies are poised to resolve the cellular heterogeneity of the bone marrow immune niche. Rather than averaging signals from bulk fecal or serum samples, spatially resolved transcriptomics and mass cytometry can map the topographical distribution of Th17/Treg clusters, myeloid progenitors, and mesenchymal stromal cells in relation to microbial metabolite gradients. This level of resolution is essential for distinguishing causative events from bystander correlates and for identifying rare but functionally dominant cell subsets that orchestrate osteoclastogenesis within the marrow microenvironment (Gui et al., 2025). Third, artificial intelligence and causal inference frameworks—such as longitudinal mediation analysis with time-varying exposures, and bidirectional Mendelian randomization with multi-step mediation—could move beyond static associations to establish temporal ordering and quantitative effect sizes across the dysbiosis–inflammation–bone loss cascade (Guan et al., 2025; Zhu et al., 2025). The development of “digital twin” models incorporating individual metagenomic, metabolomic, and immune trajectories holds promise for simulating personalized responses to probiotics or FMT before actual administration, thereby enabling iterative optimization of intervention protocols. Fourth, next-generation bio-therapeutics—including engineered bacteriophages that selectively deplete pathobionts (e.g., *Klebsiella, Escherichia-Shigella*), bacterial extracellular vesicles (BEVs) that bypass the complexity and regulatory hurdles of live probiotics while delivering active metabolites, and synthetic biology-driven microbes that sense inflammatory cues (IL-17 or TNF-α) to dynamically release anti-osteoporotic payloads (e.g., anti-RANKL nanobodies or PTH-related peptides)—represent transformative, highly targeted approaches beyond conventional supplementation (Liu H. et al., 2024; Wei et al., 2025). Collectively, these emerging directions—integrating neuroimmunology, spatial multi-omics, causal AI, and engineered live biotherapeutics—will not only resolve the mechanistic black boxes of the current axis but also pave the way for dynamically adjustable, context-aware precision medicine for OP.

Alongside these frontier directions, the synergy between GM-targeted interventions and conventional pharmacotherapy represents a more immediately translatable opportunity. Bisphosphonates and denosumab paradoxically induce GM dysbiosis, and adjuvant probiotics can restore SCFA production and mitigate rebound bone loss (Kendler et al., 2022). Teriparatide exhibits an absolute requirement for butyrate, establishing butyrogenic probiotics as “sensitizing” adjuvants to enhance anabolic efficacy, particularly in poor responders (Li et al., 2020). Romosozumab may be modulated by secondary BAs via FXR/TGR5 cross-talk with Wnt signaling, though this requires rigorous testing (Liu et al., 2025). These observations argue for factorial-design clinical trials with co-primary endpoints (BMD, fractures, SCFAs, Th17/Treg, Wnt10b) and baseline metagenomic profiling to validate drug–microbe synergy and enable responder pre-stratification. Moreover, emerging evidence identifies three reproducible microbiome endotypes in PMO—butyrate-deficient (E1), LPS-high barrier-disrupted (E2), and BA-dysregulated Th17-driven (E3)—each predicting distinct responsiveness to probiotics, prebiotics, or FMT [231]. Host genetic variants in TLR2 (rs5743708), NOD2, and tight-junction genes (OCLN, TJP1) further modulate individual responses, underscoring the need for genotype-guided stratification (Guan et al., 2025; Zhu et al., 2025). Machine learning models integrating baseline metagenomics, serum cytokine panels, and fecal SCFA concentrations can now predict clinically meaningful BMD gains with approximately 80% accuracy (Guo et al., 2026), offering a roadmap toward response-predictive prescription.

In summary, the gut-immune-bone axis has fundamentally reshaped our understanding of OP pathogenesis, transitioning the field from a bone-centric to a systems-level perspective that encompasses microbial ecology, immune homeostasis, and skeletal remodeling. Resolving subtype-specific heterogeneity, establishing causality, and translating these mechanistic insights into biomarker-stratified, microbiome-targeted interventions will be essential for developing safer and more effective anti-OP strategies. The convergence of multi-omics technologies, artificial intelligence, and precision therapeutics holds the promise of transforming OP management from a reactive, one-size-fits-all model to a proactive, personalized paradigm—addressing the global public health burden of this debilitating disease at its roots.

## 11 Conclusion

In conclusion, the gut-immune-bone axis represents a novel regulatory network underlying OP. It highlights the essential roles of intestinal microecology and the immune system in bone metabolism. A deeper understanding of this axis will help elucidate the pathogenesis of OP and provide new theoretical bases and intervention targets for developing safer and more effective anti-OP drugs and strategies, which hold important clinical value and application prospects for addressing the global public health challenge of OP.

## Glossary

AhR: Aryl Hydrocarbon Receptor
AKT: Protein Kinase B
AP-1: Activator Protein-1
BA: Bile Acid
BMD: Bone Mineral Density
BMP: Bone Morphogenetic Protein
Bregs: Regulatory B Cells
CCL: Chemokine (C-C Motif) Ligand
CCR: C-C Chemokine Receptor
CXCL: C-X-C Motif Chemokine Ligand
DCs: Dendritic Cells
DKK-1: Dickkopf-Related Protein 1
ERK: Extracellular Signal-Regulated Protein Kinase
FMT: Fecal Microbiota Transplantation
Foxp3: Forkhead Box Protein P3
FXR: Farnesoid X Receptor
GM: Gut Microbiota
IFN: Interferon
IL: Interleukin
JAK: Janus Kinase
JNK: c-Jun N-Terminal Kinase
LPS: Lipopolysaccharide
MAPK: Mitogen-Activated Protein Kinase
MHC-II: Major Histocompatibility Complex Class II
MSCs: Mesenchymal Stem Cells
NF-κB: Nuclear Factor-κB
NFATc1: Nuclear Factor of Activated T Cells 1
NK: Natural Killer
NLRP3: NOD-like receptor thermal protein domain associated protein 3
OB: Osteoblast
OC: Osteoclast
OP: Osteoporosis
OPCs: osteoclast precursor cells
OPG: Osteoprotegerin
OVX: Ovariectomy
PDGF: Platelet-Derived Growth Factor
PI3K: Phosphoinositide 3-Kinase
PMO: Postmenopausal Osteoporosis
PTH: Parathyroid Hormone
RANKL: Receptor Activator of Nuclear Factor-κB Ligand
ROS: Reactive Oxygen Species
RORγt: Retinoic Acid-Related Orphan Receptor γt
Runx2: Runt-Related Transcription Factor 2
S1PR: Sphingosine-1-Phosphate Receptor
SCFAs: Short-Chain Fatty Acids
SOST: Sclerostin
STAT: Signal Transducer and Activator of Transcription
TGF-β: Transforming Growth Factor-β
Th17: T Helper 17 Cells
TNF-α: Tumor Necrosis Factor-α
TRAP: Tartrate-Resistant Acid Phosphatase
Treg: Regulatory T Cells.
