# Supplementary material for: Gut microbiota-immune-bone axis in osteoporosis: mechanisms and intervention strategies
Source: Front Pharmacol. 2026 Oct 9;17:1879472. doi: 10.3389/fphar.2026.1879472 (PMC13692714; doi:10.3389/fphar.2026.1879472)
Supplement: Supplementary file 1 [file Supplementaryfile1.docx]

**Literature search strategy**

The research questions are as follows: (1) How do immune cells and their derived cytokines regulate the balance between osteoblasts and osteoclasts in the pathogenesis of osteoporosis? (2) What are the regulatory effects of gut microbiota and its metabolites (e.g., SCFAs, bile acids, LPS, tryptophan metabolites, TMAO) on bone metabolism via the immune system? (3) What is the mechanistic framework of the gut–immune–bone axis, and what are the efficacy and translational potential of microbiota-targeted intervention strategies (probiotics, prebiotics, fecal microbiota transplantation, and dietary regulation) for osteoporosis prevention and treatment?

The prospective protocol for this review was pre-established, with search terms and inclusion/exclusion criteria carefully selected to ensure comprehensive coverage of all relevant literature. A systematic literature search was performed on Apr 12, 2026, across the following electronic databases: Web of Science (n =186), PubMed (n =67), Embase (n=318), and China National Knowledge Infrastructure (CNKI) (n =174).

The search strategy employed free-text terms using the following keywords and Boolean operators:

(“Gut microbiota” OR “Gut microflora” OR “Gastrointestinal microbiome” OR “Microbiome, Gastrointestinal” OR “Enteric microbiota” OR “Intestinal flora”) AND (“Osteoporosis” OR “Osteoporosis, Age-Related” OR “Age-Related Osteoporosis” OR “Bone Loss, Age-Related” OR “Bone Losses, Age-Related” OR “Osteoporosis, Senile” “Osteoporoses, Senile” OR “Senile Osteoporoses” OR “Senile Osteoporosis”) AND (“Immunity” OR “Immune Response” OR “Immune Responses” OR “Response, Immune” OR “Immune Process” OR “Immune Processes” OR “Process, Immune”). External limitations were applied to each database, though specific constraints varied due to differences in search options.

No language restrictions were applied, but the search was primarily limited to studies published within the last 10 years to ensure currency of the findings. A limited number of older references of significant relevance were also included. The search strategies were adapted as necessary to accommodate the specific search interfaces and syntax requirements of each database.

**Inclusion criteria were:** (1) original research articles (in vitro, in vivo, or clinical studies) or peer‑reviewed reviews that address the roles of gut microbiota, immune cells, or their metabolites in the regulation of bone metabolism and osteoporosis; (2) studies investigating the mechanisms of the gut–immune–bone axis, including but not limited to T cell (Th17/Treg) differentiation, B cell and Breg function, macrophage (M1/M2) polarization, dendritic cell, neutrophil, mast cell, and natural killer cell involvement; (3) studies evaluating microbiota‑targeted interventions (probiotics, prebiotics, fecal microbiota transplantation, or dietary modifications) for osteoporosis prevention or treatment.

**Exclusion criteria were:** (1) studies not related to gut microbiota, immune regulation, or bone metabolism (n = 19); (2) reviews, meta‑analyses, conference abstracts, letters, editorials, and expert opinions (n = 413); (3) studies with insufficient data, duplicate publications, or those using non‑mammalian models without direct relevance to human osteoporosis (n = 1). Ultimately, 78 studies were included in this review due to their direct relevance to the gut–immune–bone axis and osteoporosis intervention strategies.

**Literature screening and data extraction**

All retrieved records were imported into reference management software for deduplication. MH and BX independently screened the titles and abstracts of the remaining records against the eligibility criteria. Full texts of potentially relevant studies were then retrieved and assessed independently by the same two reviewers. Disagreements were resolved through discussion or consultation with a third reviewer.

Data were extracted from the included studies, focusing on the interplay between gut microbiota (composition, diversity, and metabolites) and host immune responses (immune cell subsets, cytokine networks, and immunoregulatory pathways), with particular emphasis on how microbiota–immune crosstalk modulates bone metabolism and contributes to osteoporosis pathogenesis. A detailed screening flowchart is presented in **Figure 1**.

**Identification of studies via database**

Records identified from Databases (n = 764)

**Identification**

Records after duplicates removed (n =614)

Records excluded (n = 103)

Records screened (n = 614)

**Screening**

Full-text articles excluded, with reasons:

(1) studies not related to gut microbiota, immune regulation, or bone metabolism (n = 19);

(2) reviews, meta‑analyses, conference abstracts, letters, editorials, and expert opinions (n = 413);

(3) studies with insufficient data, duplicate publications, or those using non‑mammalian models without direct relevance to human osteoporosis (n = 1).

Full-text articles assessed for eligibility (n = 511)

Articles included in review (n = 78)

**Included**

**Supplementary Figure 1 Flowchart of the article selection process**

A total of 764 articles were initially identified from PubMed (n=67), Web of Science (n=), Embase (n=), and China National Knowledge Infrastructure (CNKI) (n=). After removing duplicates, 614 records were screened. Following title and abstract screening, 511 full-text articles were assessed for eligibility. Exclusion criteria applied at the full-text stage included: Ultimately, 78 studies met the inclusion criteria and were included in the review.
